# Modulation of immune responses to liposomal vaccines by intrastructural help

**DOI:** 10.1016/j.ejpb.2023.10.003

**Published:** 2023-10-04

**Authors:** Dominik Damm, Ehsan Suleiman, Jannik T. Wagner, Stephan Klessing, Felix Pfister, Hassan Elsayed, Bernd Walkenfort, Jule Stobrawe, Julia Mayer, Elisabeth Lehner, Sandra M. Müller-Schmucker, Mike Hasenberg, Richard T. Wyatt, Karola Vorauer-Uhl, Vladimir Temchura, Klaus Überla

**Affiliations:** a Institute of Clinical and Molecular Virology, University Hospital Erlangen, Friedrich-Alexander University Erlangen-Nürnberg, 91054 Erlangen, Germany; b Polymun Scientific Immunbiologische Forschung GmbH, 3400 Klosterneuburg, Austria; c Department of Otorhinolaryngology, Head and Neck Surgery, Section of Experimental Oncology and Nanomedicine (SEON), University Hospital Erlangen, 91054 Erlangen, Germany; d Department of Microbial Biotechnology, Biotechnology Research Institute, National Research Centre, Egypt; e Imaging Center Essen (IMCES), Electron Microscopy Unit (EMU), Faculty of Medicine, University of Duisburg-Essen, 45147 Essen, Germany; f Department of Biotechnology, University of Natural Resources and Life Sciences, 1190 Vienna, Austria; g The Scripps Research Institute, La Jolla, CA 92037, USA

**Keywords:** T helper liposomes, Env trimer, Peptide encapsulation, Intrastructural help, HIV vaccine, Functionalization, Nanoparticle, IgG subtype

## Abstract

The encapsulation of HIV-unrelated T helper peptides into liposomal vaccines presenting trimers of the HIV-1 envelope glycoprotein (Env) on the surface (T helper liposomes) may recruit heterologous T cells to provide help for Env-specific B cells. This mechanism called intrastructural help can modulate the HIV-specific humoral immune response. In this study, we used cationic T helper liposomes to induce intrastructural help effects in a small animal model. The liposomes were functionalized with Env trimers by a tag-free approach designed to enable a simplified GMP production. The pre-fusion conformation of the conjugated Env trimers was verified by immunogold electron microscopy (EM) imaging and flow cytometry. The liposomes induced strong activation of Env-specific B cells *in vitro*. In comparison to previously established anionic liposomes, cationic T helper liposomes were superior in CD4+ T cell activation after uptake by dendritic cells. Moreover, the T helper liposomes were able to target Env-specific B cells in secondary lymphoid organs after intramuscular injection. We also observed efficient T helper cell activation and proliferation in co-cultures with Env-specific B cells in the presence of cationic T helper liposomes. Mouse immunization experiments with cationic T helper liposomes further revealed a modulation of the Env-specific IgG subtype distribution and enhancement of the longevity of antibody responses by ovalbumin- and Hepatitis B (HBV)-specific T cell help. Thus, clinical evaluation of the concept of intrastructural help seems warranted.

## 1. Introduction

After almost four decades of continuing HIV-1 vaccine research with numerous phase-3 clinical trials that failed to provide protection, bold and novel strategies are needed to tackle the immune escape mechanisms of the virus [1–6]. Most of the previous clinical trials that focused on the elicitation of a B cell-mediated humoral immune response included either vector-encoded or proteinaceous, monomeric gp120 Env subunits and resulted in no or inefficient vaccine efficacy [7,8]. Monomeric Env subunits are known to display a multitude of immunodominant, non-neutralizing epitopes [9,10]. Current clinical studies include soluble gp140 Env trimers that were stabilized in a native-like conformation by the recombinant insertion of point mutations and flexible glycine/serine linkers (NCT03816137, NCT03699241). However, even those state-of-the-art trimeric antigens have a number of limitations, such as the induction of antibody responses to immunodominant epitopes at the artificial and non-glycosylated trimer base and a poor long-term kinetic of humoral immune responses after immunization [11–13].

Nanoparticles are an adaptable platform for vaccination [14–16]. Their size and plasticity allows them to enter the bloodstream and lymphatic vessels [17]. Biomolecular cargo can be encapsulated to protect it from a premature degradation and the nanoparticle surface may be functionalized with either antigens or targeting molecules [18]. It was independently reported that various nanoparticulate platforms functionalized with an array of Env spikes were superior in the *in vitro* activation of antigen-specific B cells and resulted in higher Env-antibody levels and a prolonged longevity of humoral immune responses upon immunization *in vivo* [19–21]. Additionally, the orthogonal conjugation of nanoparticles to C-terminal residues of native-like, stabilized Env trimers may mask immunodominant base epitopes [22].

In the past, we utilized an immunological mechanism called intrastructural help (ISH) to modulate HIV-1 Env-specific humoral immune responses [23,24]. The induction of ISH requires i) T helper nanoparticles that display Env trimers on the surface and encapsulate heterologous peptides and ii) pre-existing T cells specific for the encapsulated T helper cell epitopes. In general, after B cell receptor (BCR)-dependent uptake of T helper nanoparticles by Env-specific B cells, the encapsulated T helper peptides are presented on major histocompatibility complex class II (MHC-II) proteins. Hereby, pre-existing non-cognate T cell responses recognize their target epitope and provide help for the Env-specific B cells [25,26]. The induction of ISH may increase Env-specific antibody levels and alter the ratio of the IgG subtype responses leading to modified Fc effector functions [27]. While initial *proof-of-principle* trials were performed with lentiviral T helper virus-like particles (VLPs), we established the usage of synthetic nanoparticles (inorganic calcium phosphate nanoparticles and organic liposomes) and the encapsulation of T helper peptides that are derived from licensed vaccines (i.e. Tetanol^®^pur) for the translation of the ISH concept into a potential clinical application [23,24]. Thus, the HIV-1 immune response could be modified in humans with a T helper nanoparticle vaccine that reactivates T cell responses from childhood vaccinations. T helper epitopes from vaccine antigens against various pathogens are currently screened for applicability in ISH approaches. The concept of ISH, however, is not restricted to a HIV-related modulation of the immune response.

Hills *et al.* previously observed the improvement of immune responses against *Plasmodium falciparum* in mice using liposomes that displayed the circumsporozoite protein (CSP) and encapsulated OVA_323–339_ (OT2) peptides derived from ovalbumin (OVA) as a model epitope [28]. In a previous study, we designed and evaluated anionic T helper liposomes conjugated with Env trimers by tag-based interactions [29]. However, the presence of recombinant tags may be critical for Good Manufacturing Practice (GMP) licensing.

Thus, in the current study we functionalized cationic T helper liposomes with Env trimers via a tag-free approach and compared them to anionic equivalents in terms of uptake by antigen-presenting cells (APCs) and MHC-II presentation. The cationic T helper liposomes were further tested for their *in vivo* stability after intramuscular injection by adoptive transfer experiments. First, we reproduced the ISH results from Hills *et al.* (2016 [28]) with the focus on modulation of the HIV immune response with OT2 as a model T helper epitope. Secondly, we screened a peptide library spanning the complete HBV S antigen (HBsAg) to identify novel licensed vaccine-derived peptide epitopes for the induction of ISH. Finally, mice were primed with HBsAg-encoding DNA and boosted with T helper liposomes encapsulating a HBsAg-derived peptide. The study presents a way to improve HIV-specific humoral immune responses by a polyfunctional liposomal vaccine platform applicable for vaccination strategies against other pathogens as well.

## 2. Material and methods

### 2.1. Ethical statement, immunizations, blood collection

This study included female wildtype mice (C57BL/6NRj [BL6], Janvier, Le Genest-Saint-Isle, France; BALB/c, Charles River, Cologne, Germany) as well as in-house bred T and B cell receptor-transgenic mice of both sexes (OT2-specific mice and HIV-1 Env-specific PGT121 mice kindly provided by Dr. Diana Dudziak and Dr. Michel Nussenzweig, respectively). All mice were accommodated in ventilated cages at the animal facility (Franz-Penzoldt-Center) of the Faculty of Medicine, FAU (Erlangen, Germany) and handled as prescribed by the Federation of European Laboratory Animal Science Association and as approved by the Government of Lower Franconia (license 55.2–5323-2–154 and 55.2–2532-2–645).

Mice were 6 – 8 weeks old at the time of the first immunization. All immunizations were performed intramuscularly (i.m.) in both hind legs under constant isoflurane (CP-Pharma, Burgdorf, Germany) anesthesia. The injection volume per leg did not exceed 30 μL. A total of 30 μg DNA diluted in phosphate-buffered saline without bivalent cations (PBS) was applied i.m. in shaved injection sites using a patch bearing four electrode needles (2.5 mm spacing) with the injection needle in the center (TRi-Grid electrode array, Ichor Medical, San Diego, CA, USA) immediately followed by an electrical pulse of 63 V amplitude and 40 ms duration. Particle immunizations were normalized to the bulk amount of Env delivered with liposomes or VLPs per mouse. Mice were always immunized with a total of 300 ng Env on VLPs. The amount of Env delivered with liposomes is indicated for each experiment below. For peptide vaccinations each mouse was immunized with a total of 20 μg peptide diluted in 40 μL of a 1:1 (v/v) suspension of PBS and TiterMaxGold^®^ (TMG, Merck, Darmstadt, Germany).

Blood samples were taken under isoflurane anesthesia from the retrobulbar venous plexus using non-heparinized glass capillaries (mini-caps^®^, Hirschmann, Eberstadt, Germany). Blood was collected in BD Microtainer^®^ collection tubes (Becton Dickinson, Franklin Lakes, NJ, USA) and centrifuged for 5 min at 5000 rpm. The serum fractions were isolated and stored at −20 °C. All mice were euthanized after the last blood collection by cervical dislocation in accordance with ethical guidelines.

### 2.2. Preparation of nanoparticles

Purified conSOSL.UFO.664 trimers (SUFO.664 [30]) were kindly provided by Polymun Scientific GmbH, Klosterneuburg, Austria. Env trimers derived from the HIV-1 isolate JRFL were purified as described elsewhere [31]. The following peptides were used as T helper cell epitopes for the encapsulation into liposomes: i) The OVA-derived OT2 peptide (ISQAVHAAHAEINEAGR, piCHEM, Grambach, Austria); ii) a HBsAg-derived peptide (HBsAg_17–40_, AGFFLLTRILTIPQSLDSWWTSLN, EMC microcollections, Tübingen, Germany); iii) the Tetanus Toxoid-derived peptide p30 (FNNFTVSFWLRVPKVSASHLE, peptides & elephants GmbH, Hennigsdorf, Germany); iv) a library (n = 22) of 20-mer peptides with 10 amino acid (aa) overlaps spanning the whole HBsAg aa sequence (GenScript Biotech, Piscataway, NJ, USA).

#### 2.2.1. Cationic T helper liposomes

Carboxyl-functionalized, peptide-loaded liposomes were prepared by thin-film hydration as described elsewhere [32,33]. Liposomes were composed of 81 mol% 1,2-dioleoyl-*sn*-glycero-3-phosphocholine (DOPC, Lipoid GmbH, Ludwigshafen am Rhein, Germany), 15 mol% 1,2-dioleoyl-3-trimethylammonium-propane [chloride salt] (DOTAP, Merck KGaA, Darmstadt, Germany) and 4 mol% monodisperse 1,2-distearoyl-*sn*-glycero-3-phosphoethanolamine-*N*-[carboxy(polyethylene glycol)] with 14 polyethylene glycol units (DSPE-PEG_14_-COOH, Biochempeg Scientific Inc., Watertown, MA, USA). The positive zetapotential of the nanoparticles is based on the presence of DOTAP in the formulation, while the terminal COOH groups are used for covalent conjugation of tag-free, non-functionalized Env trimers as described in detail in Suleiman *et al.* (2020) [33]. Briefly, a 4-fold molar excess of *N*-Hydroxysulfosuccinimide sodium salt (Sulfo-NHS) and a 10-fold molar excess of *N*-(3-Dimethylaminopropyl)-*N*′-ethylcarbodiimide hydrochloride (EDC, both Merck KGaA, Darmstadt, Germany) over the accessible carboxyl groups on the surface of the liposomes were used for activation. Activation was performed in 5 mM phosphate buffer (pH 6.0 with 15 mM NaCl and 270 mM sucrose) at a total lipid concentration of 21.2 mM (424 μM accessible carboxyl-functionalized lipid). The reaction was then allowed to proceed for 15 min at 25 °C and 1,400 rpm. Excess EDC was chemically inactivated by addition of β-mercaptoethanol. The activated liposomes were then further diluted and mixed with tag-free Env trimers to give a final concentration of 10 mM total lipid (200 μM accessible carboxyl-functionalized lipid) and 706 nM (254 μg/mL) Env trimer. This corresponds to a 94-fold molar excess of accessible carboxyl groups over the three C-termini of trimeric Env. It was previously shown that a minimum of 9 mol% DOTAP in the liposomal formulation is crucial to attract negatively charged Env trimers [33]. The conjugation reaction was then allowed to proceed for 2 h at 25 °C and 1400 rpm. The quenching, purification, concentration and characterization were performed as previously described for anionic T helper liposomes [29]. Cationic T helper liposomes used in this study were surface-functionalized with tag-free SUFO.664 trimers and encapsulated either HBsAg_17–40_ (Env-Lipo-HBV) or OT2 (Env-Lipo-OVA) peptides.

#### 2.2.2. Anionic T helper liposomes

Anionic liposomes used in this study were produced and purified as previously described [29]. The nanoparticles encapsulated OT2 and were conjugated with His-tagged SUFO.664 trimers in a tag-mediated two-step coupling mechanism.

#### 2.2.3. Neutral T helper liposomes

T helper liposomes with a neutral zetapotential were produced and purified as previously described [29]. In contrast to the charged liposomes, the nanoparticle surface was functionalized with stabilized JRFL Env trimers via a mechanism described by Ingale *et al.* (2016) and Bale *et al*. (2017) [20,22] and either OT2 or Tetanus Toxoid-derived p30 peptides were encapsulated by passive inclusion during hydration of the lipid film [29].

#### 2.2.4. Lentiviral T helper VLPs

T helper VLPs incorporating OT2 or HBsAg peptides and displaying membrane-embedded conSOSL.UFO.750 trimers (SUFO.750 [30]) were produced and quantified as previously described [29]. In short, HEK293T cells were co-transfected with plasmids encoding SUFO.750 and a HIV-GagPol/peptide fusion construct. The VLPs were pelleted from the cell supernatants by ultracentrifugation through a 35 % sucrose cushion and resuspended in PBS. The concentration of Env and Gag in VLP preparations was determined by enzyme-linked immunosorbent assay (ELISA).

### 2.3. Isolation and analysis of primary murine cells

The purification of primary B cells, T cells, dendritic cells (DCs) and lymphocytes from isolated organs or tissue as well as activation and proliferation of these cells in different *in vitro* assays were performed as previously described with slight modifications in the antibody panel [24,29]. In this study, all flow cytometry measurements were performed with an Attune NxT Flow Cytometer (Thermo Fisher, Waltham, MA, USA).

#### 2.3.1. In vitro B cell activation

After 24 h incubation in the presence of nanoparticles, the activation of B cells was analyzed by immunostaining for B cell activation markers (CD69, CD80, CD86), for B cell identification marker CD19 and for living cells (Fixable Viability Dye). The staining was done using anti-CD80-FITC (16–10A1) and Fixable Viability Dye eFluor450 (both eBioscience^™^, Thermo Fisher, Waltham, MA, USA), anti-CD86-PE-Cy7 (GL1) and anti-CD69-BV510 (H1.2F3, both Becton Dickinson, Franklin Lakes, NJ, USA) as well as anti-CD19-PerCP (6D5, BioLegend, San Diego, CA, USA).

#### 2.3.2. Adoptive transfer and B cell proliferation

Env-specific PGT121 B cells were isolated from transgenic mice and labelled in the presence of 21 μM carboxyfluorescein succinimidyl ester (CFSE, Thermo Fisher, Waltham, MA, USA) as a proliferation tracer. An average of 6 × 10^6^ CFSE+ B cells in a total volume of 100 μL PBS were intravenously injected in the tail veins of wildtype (*wt*) BL6 mice. After 18 h, the mice were immunized i.m. in both hind legs with either soluble SUFO.664 trimers or cationic T helper liposomes normalized to 3 μg Env per animal. Mice that received the adoptive B cell transfer, but no further injection, were used as mock control. The mice were sacrificed after 72 h. The spleens and inguinal lymph nodes were isolated. The splenocytes were further processed by magnetic cell separation (MACS) to purify the splenic B cells. The lymphocytes and splenic B cells were then stained with anti-CD45R/B220-APC (RA3–6B2, Becton Dickinson, Franklin Lakes, NJ, USA). Finally, CFSE+ B cells were analyzed for proliferation.

#### 2.3.3. T helper cell activation and proliferation in co-cultures with APCs

OT2-specific CD4+ T cell proliferation in co-cultures with wildtype DCs incubated in the presence of liposomal formulations normalized to the bulk concentration of encapsulated OT2 peptide (Fig. S2) was evaluated as previously described [29]. *In vitro* ISH effects were assessed by co-cultures of OT2-specific T cells and Env-specific B cells (1 × 10^5^ cells per well, respectively) incubated with T helper liposomes in different dilutions normalized to the bulk concentration of Env (Fig. 2) as described elsewhere with minor modifications [29]. We evaluated the activation of the CD4+ T cells after 24 h via flow cytometry by surface staining with T cell marker anti-CD4-SB600 (clone RM4–5) as well as activation markers anti-CD69-PE-Cy7 (clone H1.2F3) and anti-CD44-FITC (clone IM7, all eBioscience, Waltham, MA, USA). A live/dead staining with Fixable Viability Dye eFluor450 (Thermo Fisher, Waltham, MA, USA) was done in parallel. For the assessment of T cell proliferation, OT2 T cells previously labelled in the presence of 7 μM CFSE were used in the co-culture assay. After 72 h, the cells were stained with anti-CD4-SB600 and Fixable Viability Dye and measured by flow cytometry. Either wildtype T helper cells or T helper liposomes that encapsulated HBsAg peptides were used in control samples.

#### 2.3.4. Cytokine profiling in T helper cells from immunized mice

We analyzed the cytokine secretion pattern of epitope-specific T helper cells two weeks after the last immunization by intracellular cytokine staining (ICS). A total of 1 × 10^6^ splenocytes from immunized mice were seeded per well into U-bottom 96-well microplates (Greiner Bio-One, Kremsmünster, Austria). For antigen restimulation, the splenocytes were incubated with 5 μg/mL peptide in the presence of 2 μg/mL anti-mouse CD28 antibody and 3 μg/mL Brefeldin A (both eBioscience, San Diego, CA, USA) over night at 37 °C and 5 % CO_2_. After incubation, the cells were washed with MACS buffer (1 % bovine serum albumine (BSA), 1 mM 2,2′,2′’,2′’’-(Ethane-1,2-diyldinitrilo)tetraacetic acid [EDTA] in PBS) and stained with a CD4 antibody (anti-mouse CD4-SB600 [RM4–5], eBioscience, San Diego, CA, USA) and Fixable Viability Dye eFluor450 (Thermo Fisher Scientific, Waltham, MA, USA). Finally, the cells were fixated with 2 % paraformaldehyde in MACS buffer and permeabilized using 0.5 % Saponin in MACS buffer. For ICS, fluorescently labelled antibodies specific for interleukin-2 (IL-2), interferon-gamma (IFN-γ) and tumor necrosis factor alpha (TNF-α) (anti-mouse IL-2-APC [JES6–5H4], IFN-γ-PE [XMG1.2], TNF-α-PE-Cy7 [MP6-XT22], eBioscience, San Diego, CA, USA) were added. After 20 min staining on ice, the cells were washed twice with permeabilization buffer and twice with MACS buffer. The cytokine secretion in all CD4+ T cells was finally evaluated by flow cytometry.

### 2.4. Antibody staining of liposomes

#### 2.4.1. Immunogold EM imaging of cationic T helper liposomes

The ab201808 – GOLD Conjugation Kit (10 nm 20 OD; Abcam, Cambridge, UK) was used to couple monoclonal Env-specific antibodies with gold beads (2G12, PG19, Polymun Scientific GmbH, Klosterneuburg, Austria; 17b, PGT145, PGT121, NIH AIDS Reagent Program, Bethesda, MD, USA; RM19R kindly provided by Dr. Marit van Gils, AMC, Amsterdam, NL). For microscopic immunoelectron analysis, the respective sample suspension was first diluted 1:50 with PBS and a 7 μL drop of this dilution was then incubated on a 200 mesh Formvar^®^/carbon-coated nickel grid (#S162N; Plano, Wetzlar, Germany) for 10 min at room temperature (RT) to let liposomes adhere to the grid surface (“drop-on-grid” approach). After incubation, the excess of sample suspension was carefully removed with a triangular piece of filter paper. All following incubation steps were conducted using a “grid-on-drop” approach, in which the drop volume was 30 μL, at RT. First, the sample was washed with PBS (3× 5 min, each) before it was blocked with PBS+ (PBS + 0.5 % (w/v) BSA and 0.15 % (w/v) glycine) for 10 min. After removal of the supernatant with filter paper, the grid was placed on the respective antibody solution (adjusted to 3 ng/μL with PBS) and incubated for 12 h. The sample was then washed with deionized water (3× 5 min), before the supernatant was carefully removed with filter paper. Ultimately, the sample was negatively stained on a 10 μL drop of aqueous 1.5 % phosphotungstic acid (PTA) for 1 min. After this last incubation step, the PTA solution was gradually removed with a filter paper and the sample was air-dried prior to transmission electron microscopy (TEM) analysis.

#### 2.4.2. Analysis of liposomes by flow cytometry

The assay was performed as previously described with a slightly different antibody panel [29]: F105, PGT121, PG9, PGT145 (NIH AIDS Reagent Program, Bethesda, MD, USA); RM19R (kindly provided by Dr. Marit van Gils, AMC, Amsterdam, NL); 2G12 (Polymun Scientific GmbH, Klosterneuburg, Austria). The hCMV-specific anti-gB antibody 27–287 (kindly provided by Dr. Michael Mach, Virologisches Institut, Universitätsklinikum Erlangen, Germany) was used as an isotype control.

### 2.5. Analysis and quantification of humoral immune responses

Serum ELISAs were performed to determine the immunoglobulin G (IgG) concentration and subtype distribution in sera from immunized mice. Opaque, white Corning^®^ 96-well, flat-bottom, polystyrene, high-binding microplates (Corning Inc., Corning, NY, USA) were coated over night with 100 ng/well SUFO.664 in coating buffer (0.1 M Na_2_CO_3_, 0.1 M NaHCO_3_ in H_2_O, pH 9.6). The wells were washed and blocked for 1 h at RT with 300 μL of 5 % skimmed milk in PBS supplemented with 0.05 % Tween-20 (PBS/T). Afterwards, the blocking agent was replaced with 100 μL murine sera dilutions in 2 % skimmed milk in PBS/T. The dilution factor was the same for each serum that was tested simultaneously in one experiment and was chosen as measured by the reactivity of the sera between background and saturation levels. For quantification, 2-fold serial dilutions of recombinant b12 antibodies with murine IgG1, IgG2a, IgG2b, IgG2c or IgG3 Fc domains produced as outlined in Klessing *et. al.* (2020) were added in parallel [27]. After 1 h incubation at RT in the presence of serum dilutions and antibodies, the wells were washed thrice with PBS/T. 100 μL of horse radish peroxidase-coupled anti-murine-IgG (Dianova, Hamburg, Germany) or anti-murine IgG1, IgG2a, IgG2b, IgG2c or IgG3 (Southern Biotech, Birmingham, AL, USA) were added for 1 h at RT. All secondary antibodies were diluted 1:5000 in 2 % skimmed milk in PBS/T. The plate was washed six times with PBS/T. All ELISAs were evaluated measuring the relative light units per second immediately after addition of 60 μL ECL substrate by the Orion Microplate Luminometer (Berthold Detection Systems, Pforzheim, Germany).

### 2.6. Software

Statistical analyses were performed with the GraphPad Prism 7 software (GraphPad Software Inc., San Diego, CA, USA). Evaluation of flow cytometry data was done with FlowJo software (BD Biosciences, Franklin Lakes, NJ, USA).

## 3. Results

### 3.1. Production of T helper liposomes

**Fig. 1.**
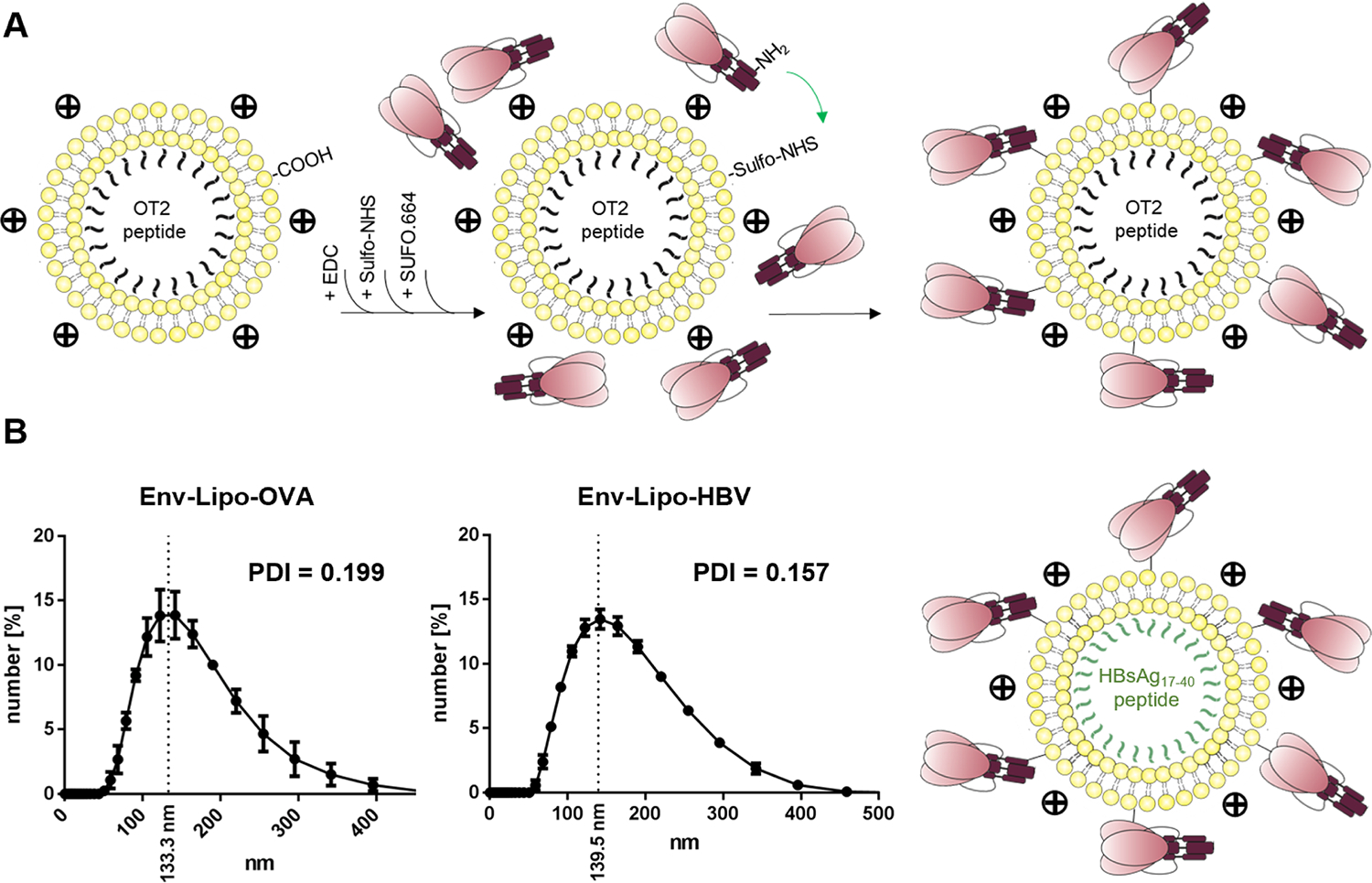
Design and production of cationic T helper liposomes. **(A)** Liposomes composed of 81 mol% DOPC, 15 mol% DOTAP and 4 mol% DSPE-PEG_14_-COOH encapsulating either OT2 or HBsAg_17–40_ peptides were produced by thin-film hydration. SUFO.664 Env trimers with a negative zetapotential were attracted to the cationic liposomal surface via electrostatic interactions. The terminal carboxyl groups of the liposomes were activated by EDC/Sulfo-NHS and subsequently used for covalent crosslinking with primary amine groups that were accessible on the Env interface leading to a liposomal surface functionalization with Env trimers. **(B)** The resulting T helper liposomes (Env-Lipo-OVA and Env-Lipo-HBV) were analyzed by DLS on a ZetaSizer instrument for their mean hydrodynamic diameter and PDI. Mean graphs ± SEM are depicted from three independent measurements.

The main characteristics of T helper nanoparticles are the encapsulation of heterologous T helper epitopes or proteins and the functionalization of the surface with the antigen of interest. Recently, we improved the design and functionality of anionic T helper liposomes by an electrostatically driven peptide encapsulation approach and the orthogonal conjugation of native-like Env trimers with C-terminal His-tags via affinity interactions with Ni(NTA)-terminated phospholipids and a covalent EDC/Sulfo-NHS-based crosslinking [29]. Since nickel-containing reagents and the usage of recombinant tags are major obstacles for GMP processing, we developed a strategy to couple native-like Env trimers without the need for a recombinant tag. We altered the lipid composition to produce liposomes with a cationic surface charge and utilized the negative zetapotential of Env trimers to bring them in close proximity with the nanoparticles. Here, a consensus S (conS)-based gp140 Env trimer which was stabilized via a flexible glycine-serine linker between the gp120 and gp41 ectodomain subunits and by the insertion of another flexible UFO linker (uncleaved pre-fusion optimized) into the gp41 H1 helix beside several point mutations was used as the antigen of choice (SUFO.664 [30]). Phospholipids with terminal carboxyl groups (DSPE-PEG_14_-COOH) were harnessed for covalent EDC/Sulfo-NHS linkage targeting primary amines on the Env trimers (Fig. 1). The coupling chemistry was shown in detail in Suleiman *et al.*, (2020) [33]. This way, we produced cationic T helper nanoparticles that encapsulated either the MHC-II-restricted OVA-derived OT2 peptide (Env-Lipo-OVA) or an HBV-derived peptide (HBsAg_17–40_, Env-Lipo-HBV). Both batches of liposomes were characterized by dynamic light scattering (DLS). Env-Lipo-OVA had a mean hydrodynamic diameter of 133.3 nm and a polydispersity index (PDI) of 0.199, while Env-Lipo-HBV were 139.5 nm in average size and showed a slightly more homogenous population with a PDI of 0.157 (Fig. 1).

The conformation of SUFO.664 Env trimers on the surface of the T helper liposomes was evaluated by immunogold EM imaging and flow cytometry as previously established for anionic T helper liposomes (Fig. S1) [29]. Our established protocol for immobilization of nanoparticles on a TEM grid had to be modified for cationic liposomes. Even though the immobilization rate was less efficient, we stained the liposomes with monoclonal Env antibodies conjugated with gold beads. These are recombinant forms of antibodies secreted from B cell clones of HIV-1 elite controllers and are widely used to define the conformation of Env trimers, since their target epitopes and binding features are well characterized [34]. We observed strong binding signals for trimer-specific apex antibodies PG9 and PGT145 (Fig. S1A). Glycan-dependent antibody 2G12 and gp120 interface antibody PGT121 both bound in a comparable manner. Importantly, the binding of trimer base-specific antibody RM19R was reduced in comparison to apex- and glycan-specific antibodies. No immunogold signals were detected after incubation with CD4i control antibody 17b that binds an open conformation of Env which does not resemble the pre-fusion state. We obtained similar binding results with the flow cytometric analysis of antibody-stained liposomes (Fig. S1B). Binding signals were most prominent with PGT121, PG9 and PGT145 antibodies. Open-conformation antibody F105 and base antibody RM19R demonstrated binding in a comparable range as the isotype control, which was a monoclonal antibody specific for the hCMV glycoprotein B (gB). A conformational ELISA that compared Env conjugated to cationic T helper liposomes and soluble Env trimers was described elsewhere [33]. In summary, the antibody binding data indicate that the native-like conformation of SUFO.664 trimers on the liposomal surface was maintained during and after the conjugation procedure.

### 3.2. Functional characterization of T helper liposomes in vitro and in vivo

In a previous study, we have established *in vitro* co-culture assays of T cells with either DCs or B cells to evaluate the uptake of the nanoparticles by these APCs and the activation and proliferation of CD4+ T cells after presentation of peptides derived from the nanoparticles on MHC-II complexes of the APCs [29]. We used these assays in the present study to compare the influence of the nanoparticle charge (zetapotential) on the efficiency of T helper cell stimulation. First, we isolated splenic DCs from BL6 mice (*wt* DCs) and co-cultured them with OT2-specific CD4+ T cells purified from the spleens of T cell receptor-transgenic mice (OT2 T cells) and labeled with CFSE. As a negative control, co-cultures were done with DCs and CFSE+ T cells from BL6 mice (*wt* T cells). The co-cultures were incubated for 72 h with either anionic or cationic Env-Lipo-OVA or with soluble OT2 peptide as a positive control in 1:3 dilutions normalized to the bulk concentration of peptide (300 – 3 ng/mL). Proliferation was measured via flow cytometry by evaluating the distribution of the proliferation marker CFSE in divided cells. Here, the proliferation of OT2 T cells was improved in samples incubated with cationic Env-Lipo-OVA compared to anionic nanoparticles (Fig. S2). As expected, no proliferation of *wt* T cells was induced, which demonstrated peptide-specific stimulation of the OT2 T cells.

**Fig. 2.**
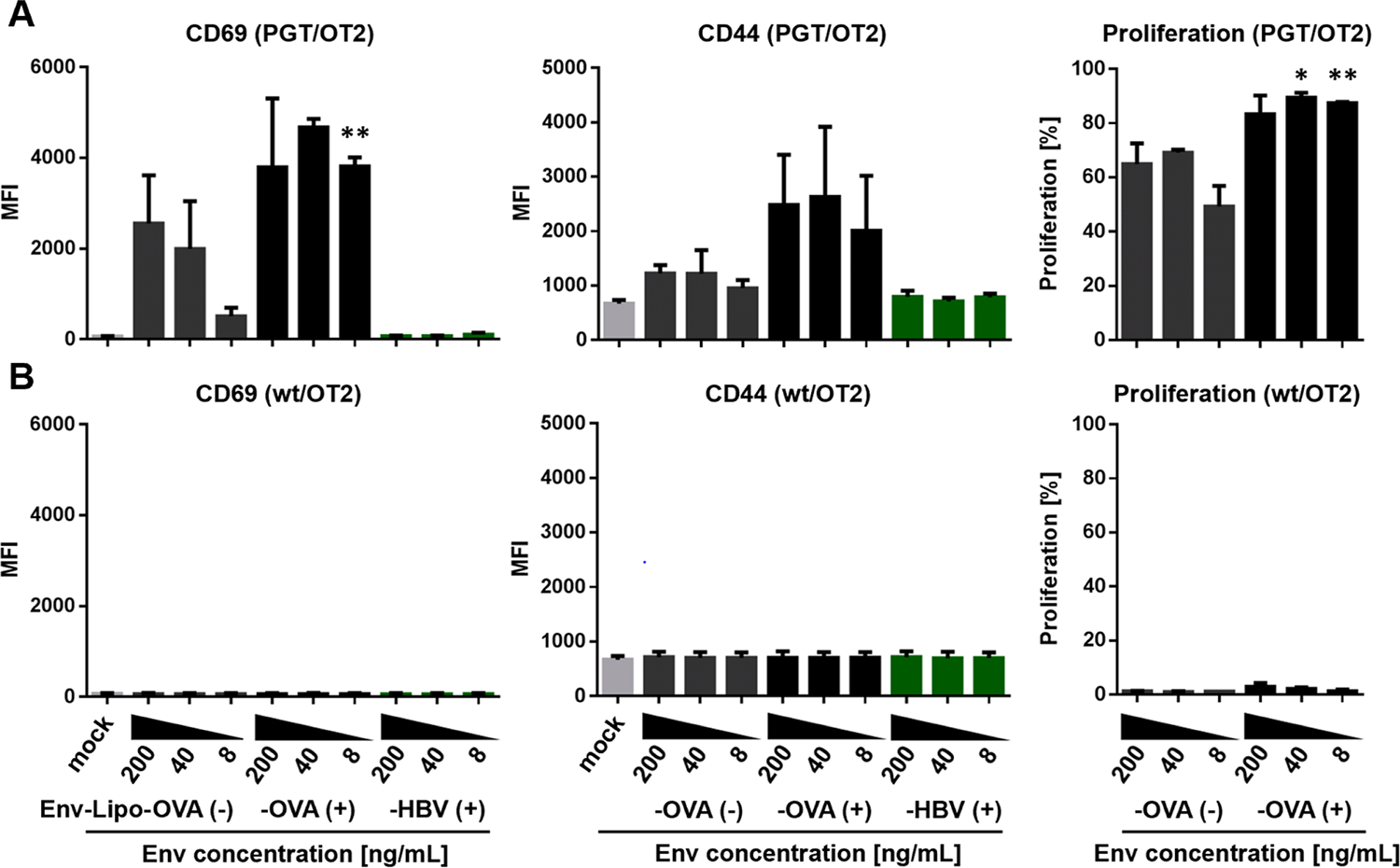
*In vitro* intrastructural help. **(A)** Co-cultures of 1 × 10^5^ Env-specific PGT121 B cells (PGT, upper panel) or **(B)** 1 × 10^5^ wildtype B cells (*wt*, lower panel) with 1 × 10^5^ OT2-specific (OT2) T helper cells were seeded in the presence of dilutions of anionic T helper liposomes encapsulating OT2 peptide [Env-Lipo-OVA(−)] or cationic T helper liposomes encapsulating OT2 or an HBsAg peptide [Env-Lipo-OVA(+), Env-Lipo-HBV(+)] normalized to the bulk concentration of Env (200 – 8 ng/mL) in the samples. Unstimulated co-cultures (mock) were used as negative controls. After 18 h the activation of living, CD4+ T cells was evaluated by flow cytometry via upregulation of T cell activation markers CD69 and CD44. On day 3, proliferation of CFSE-labeled T cells was analyzed based on the CFSE distribution pattern in the CD4+ population. The columns ± SEM represent the mean median fluorescence intensity (MFI) values of activated cells or the mean percentage of proliferated cells from three independent experiments. The activation and proliferation of CD4+ T cells in samples incubated with cationic and anionic Env-Lipo-OVA liposomes at the same bulk Env concentration were statistically compared with a parametric students T test. * *p <* 0.05; ** *p <* 0.005.

We further performed a variation of this assay with Env-specific PGT121 B cells as APCs, which express the broadly-neutralizing Env antibody PGT121 as BCR, to study the BCR-dependent uptake of the T helper liposomes and stimulation of OT2-specific CD4+ T cells (*in vitro* ISH). In this regard, the liposomal dilutions were normalized to the bulk concentration of Env (200 – 8 ng/mL). Similar to unspecific uptake by DCs, all samples incubated with cationic Env-Lipo-OVA resulted in a stronger induction of T cell activation markers CD69 and CD44 and improved T cell proliferation rates (Fig. 2) compared to anionic liposomes. In particular, CD44, a marker for TCR-specific activation, was almost at the level of the unstimulated mock control with anionic nanoparticles. No T cell activation and proliferation was seen when *wt* B cells were used, indicating a B cell receptor-dependent response. Furthermore, we did not see any effects with Env-Lipo-HBV, which proved the T cell specificity for the heterologous OT2 peptide in the assay.

Since the differences between anionic and cationic liposomes in T cell activation and proliferation were most prominent with an Env bulk concentration of 8 ng/mL, we performed a deeper analysis of the cell proliferation using the FlowJo Proliferation Platform^™^ (Fig. S3). The OT2 T helper cells in co-cultures incubated with cationic T helper liposomes proliferated more rapidly with only 2 % and 5 % of living cells remaining in the undivided generation 0 and generation 1, respectively. In contrast, 17 % undivided cells and 27 % cells in generation 1 were detected in samples treated with anionic liposomes. Based on the improved stimulation of T cells in co-cultures with APCs, we decided to move on with cationic T helper nanoparticles only.

**Fig. 3.**
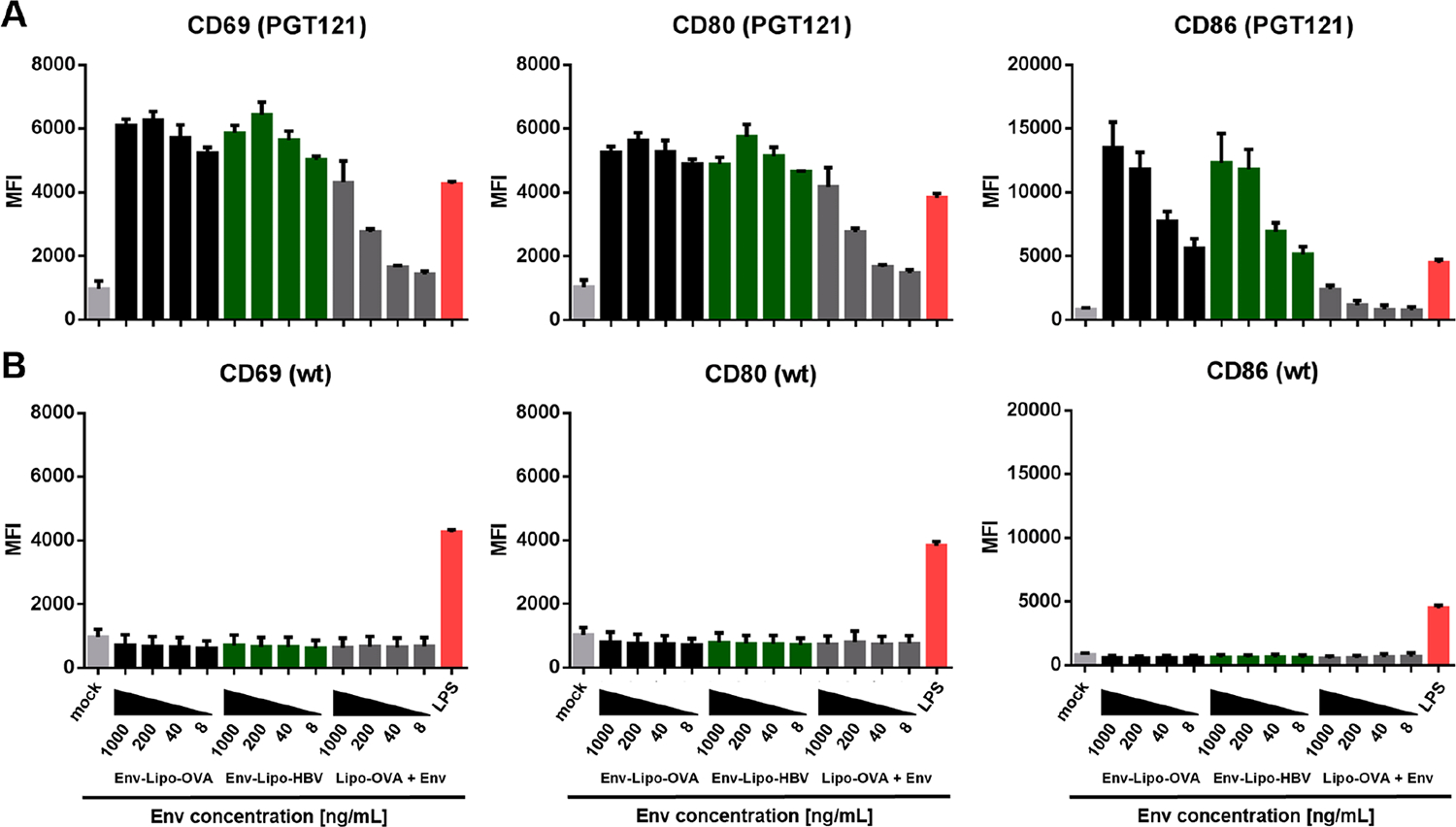
*In vitro* B cell activation by cationic T helper liposomes. **(A)** Either 2 × 10^5^ Env-specific PGT121 (upper panel) or **(B)** 2 × 10^5^ wt B cells (lower panel) were incubated for 18 h in the presence of cationic T helper liposomes (Env-Lipo-OVA or Env-Lipo-HBV) normalized to the bulk concentration of Env per sample (1000 – 8 ng/mL). B cells were treated with 1 μg/mL lipopolysaccharide (LPS) as a positive control for polyclonal B cell activation. Unstimulated (mock) samples and B cells incubated with a mixture of unconjugated OT2 liposomes and soluble SUFO.664 (Lipo-OVA + Env) were used as negative controls. B cell activation was evaluated by the upregulation of CD69, CD80 and CD86 measured via flow cytometry. The columns ± SEM represent the means of the median fluorescence intensities (MFI) of three independent experiments.

As outlined above, two types of cationic T helper liposomes, that encapsulated OVA- or HBV-derived peptides, were produced for our study. We compared the ability of both nanoparticle batches to induce B cell activation upon BCR binding in another functional *in vitro* assay (Fig. 3). PGT121 B cells were incubated in the presence of dilutions of cationic T helper liposomes normalized to the bulk concentration of Env (1000 – 8 ng/mL). As a negative control, we incubated B cells with unconjugated cationic OT2 liposomes and soluble SUFO.664 trimers (Lipo-OVA + Env). After 18 h of incubation, we measured strong and comparable upregulation of early activation markers CD69 and CD80 with both Env-Lipo-OVA and Env-Lipo-HBV. Interestingly, the nanoparticles also induced strong upregulation of activation marker CD86 with 1000 ng/mL and 200 ng/mL bulk Env, but a much less pronounced CD86 induction with lower Env concentrations.

The combination of unconjugated liposomes and soluble Env only induced a moderate upregulation of all activation markers with the highest Env concentration, which dropped to background levels with further dilutions of the nanoparticles and antigen (Fig. 3). Importantly, no activation of *wt* B cells was observed in the assay as expected, proving that the activation of PGT121 B cells in the presence of T helper liposomes was BCR-specific.

**Fig. 4.**
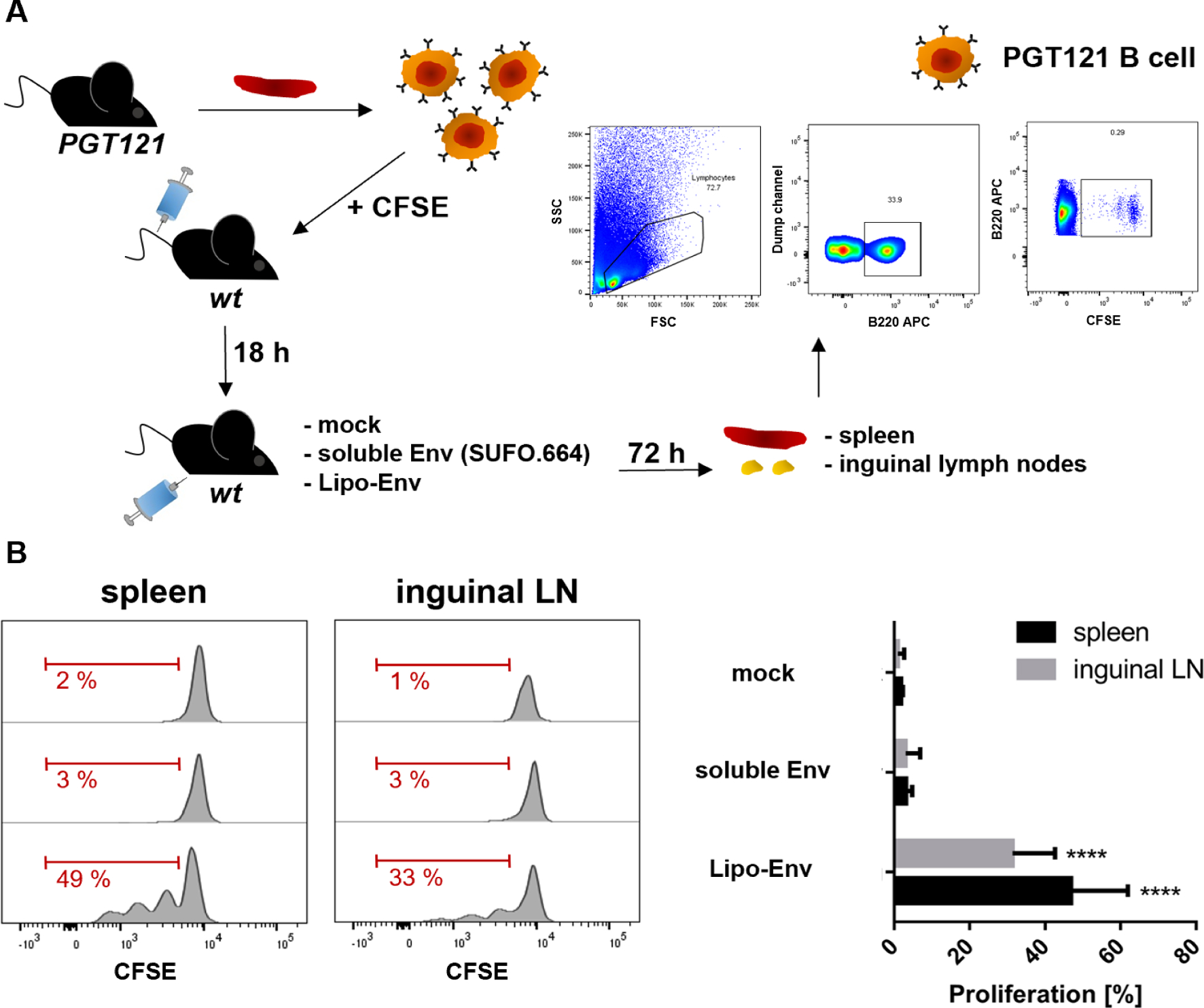
Adoptive transfer of Env-specific B cells. **(A**) Splenic B cells from PGT121 mice were purified by MACS and labeled in the presence of 21 μM CFSE. Subsequently, 6–8 × 10^6^ CFSE-labeled Env-specific B cells were intravenously injected into the tail veins of *wt* BL6 mice. After 18 h, the recipient mice were immunized i.m. with soluble SUFO.664 Env trimers (n = 4) or cationic Env-liposomes (Lipo-Env, n = 4) with a total of 3 μg Env per animal. A control group (n = 2) was immunized with buffer only (mock). 72 h later, the recipient mice were sacrificed and both spleens and inguinal lymph nodes (LN) were isolated. The splenocytes and lymphocytes were analyzed by flow cytometry. B220+ cells were gated. The CFSE-labeled Env-specific B cells were identified from this population. **(B)** Proliferating cells were gated based on the CFSE distribution pattern in the histograms. The columns ± SEM represent the means of proliferating PGT121 B cells per group and secondary lymphoid organ. Significant proliferation in the liposomal group compared to the control group was analyzed by one-way ANOVA with Tukey’s multiple comparison post-hoc test. **** *p <* 0.0001.

In the past, we investigated *in vivo* ISH effects by intramuscular application of T helper VLPs or CaPs [24,27]. We showed that VLPs mainly targeted cognate B cells in the spleen after intravenous (i.v.) application, while the targeting was restricted to the draining lymph nodes after subcutaneous (s.c.) injection [35]. Likewise, CaPs induced proliferation of cognate B cells in the draining lymph nodes after both i.m. and s.c. immunization [36]. More recently, we have demonstrated in an *ex vivo* trial that DCs isolated from the spleens and inguinal lymph nodes of mice immunized with unconjugated OT2 liposomes could stimulate OT2-specific T cells by MHC-II-mediated presentation of the nanoparticle-derived peptides [29]. However, DCs take up nanoparticles and proteins in an unspecific manner and may have encountered the liposomes at the site of injection and migrated to the secondary lymphoid organs afterwards [35]. In order to proof *in vivo* stability of T helper liposomes after i.m. immunization, we performed a B cell adoptive transfer experiment (Fig. 4). Env-specific B cells were purified from the spleens of PGT121 mice, labeled with CFSE and were injected i.v. into the tail veins of *wt* BL6 mice. 18 h later, the *wt* mice were immunized i.m. with either cationic T helper liposomes or soluble Env. A mock group remained non-vaccinated. The mice were sacrificed after 3 days and both splenocytes as well as inguinal lymphocytes were isolated and stained for the B cell marker B220. We then gated cells positive for B220 and CFSE. B cell proliferation was evaluated based on the CFSE distribution pattern (Fig. 4A). We detected Env-specific B cell proliferation in the spleen and inguinal lymph nodes only in mice that were immunized with T helper liposomes, but not with soluble Env trimers. Furthermore, the proliferation was more pronounced in the spleens than in the inguinal lymph nodes (Fig. 4B). These results strongly indicated that T helper liposomes upon i.m. injection reach the secondary lymphoid organs as functional nanoparticles, which is a crucial pre-requisite for successful induction of ISH effects.

Taken together, cationic T helper liposomes conjugated with Env trimers in a tag-independent manner demonstrated functional advantages over anionic T helper nanoparticles in terms of T helper cell stimulation after uptake by APCs. Furthermore, the nanoparticles reach the sites of secondary lymphoid organs after i.m. injection. These observations supported further *in vivo* experiments that aimed to induce a modulation of Env-specific humoral immune responses with cationic T helper liposomes via ISH.

### 3.3. In vivo induction of ISH effects with cationic T helper liposomes

As stated above, ISH effects are mediated by the recruitment of pre-existing heterologous T cell responses that provide help for B cells specific for the antigen of interest. In a potential clinical setup we would aim to stimulate T cell responses previously generated by recommended vaccinations in the childhood (i.e. against Tetanus or HBV) with our T helper nanoparticle vaccine candidates. For trials in small animal models we first induce the heterologous T cell responses by a prime immunization with licensed vaccines or DNA (i.e. Tetanol^®^pur or HBsAg-encoding plasmids) and subsequently boost with T helper nanoparticles (VLPs or synthetic nanoparticles). Other trials that act as *proof-of-principle* were also performed by the induction and recruitment of T cell responses against model antigens like HIV-1 capsid components in combination with a lentiviral VLP boost or OVA-derived OT2 peptide with OT2-encapsulating liposomes as booster immunizations [25,28].

Our initial trials to induce ISH effects in mice with synthetic nanoparticles were performed with a first generation of T helper liposomes that were described and characterized elsewhere [29]. In short, these liposomes were orthogonally conjugated with a dense array of JRFL Env spikes and T helper peptides were encapsulated by passive inclusion during liposomal formation. Dose finding experiments in mice revealed that two intramuscular immunizations with these nanoparticles in a 4-week interval resulted in significantly elevated anti-Env IgG1 responses (Fig. S4A) compared to naïve mice, when the vaccine doses were adjusted to 10 μg Env per animal. Two immunizations with 1 μg Env on liposomes did not induce any detectable immune responses. In the following, we primed mice with two shots of the licensed vaccine Tetanol^®^pur diluted in PBS or with buffer only as a control and boosted them twice with first-generation T helper liposomes encapsulating either Tetanus Toxoid-derived p30 or OT2 peptides. Surprisingly, despite the induction of anti-Env IgG1 responses, we did not see significant differences between ISH and control groups (Fig. S4B). On the contrary, a positive control group that received T helper VLPs incorporating p30 epitopes did show significantly increased Env-specific IgG1 levels, which reproduced previous results by Elsayed *et al.* (2018) [23].

From these observations we deduced the following conclusions: i) The quantitative encapsulation of T helper peptides may be crucial for the *in vivo* induction of ISH. ii) T helper liposomes must reach the secondary lymphoid organs without dissociation. iii) Lentiviral T helper VLPs act as a reliable model for ISH effects.

Thus, based on these hypotheses, we steadily improved our nanoparticle design. The cationic T helper liposomes used in this study encapsulate a high number of T helper epitopes via an electrostatically-driven approach [32], they are covalently conjugated with Env trimers (Fig. 1) and reach secondary lymphoid organs (Fig. 4).

**Fig. 5.**
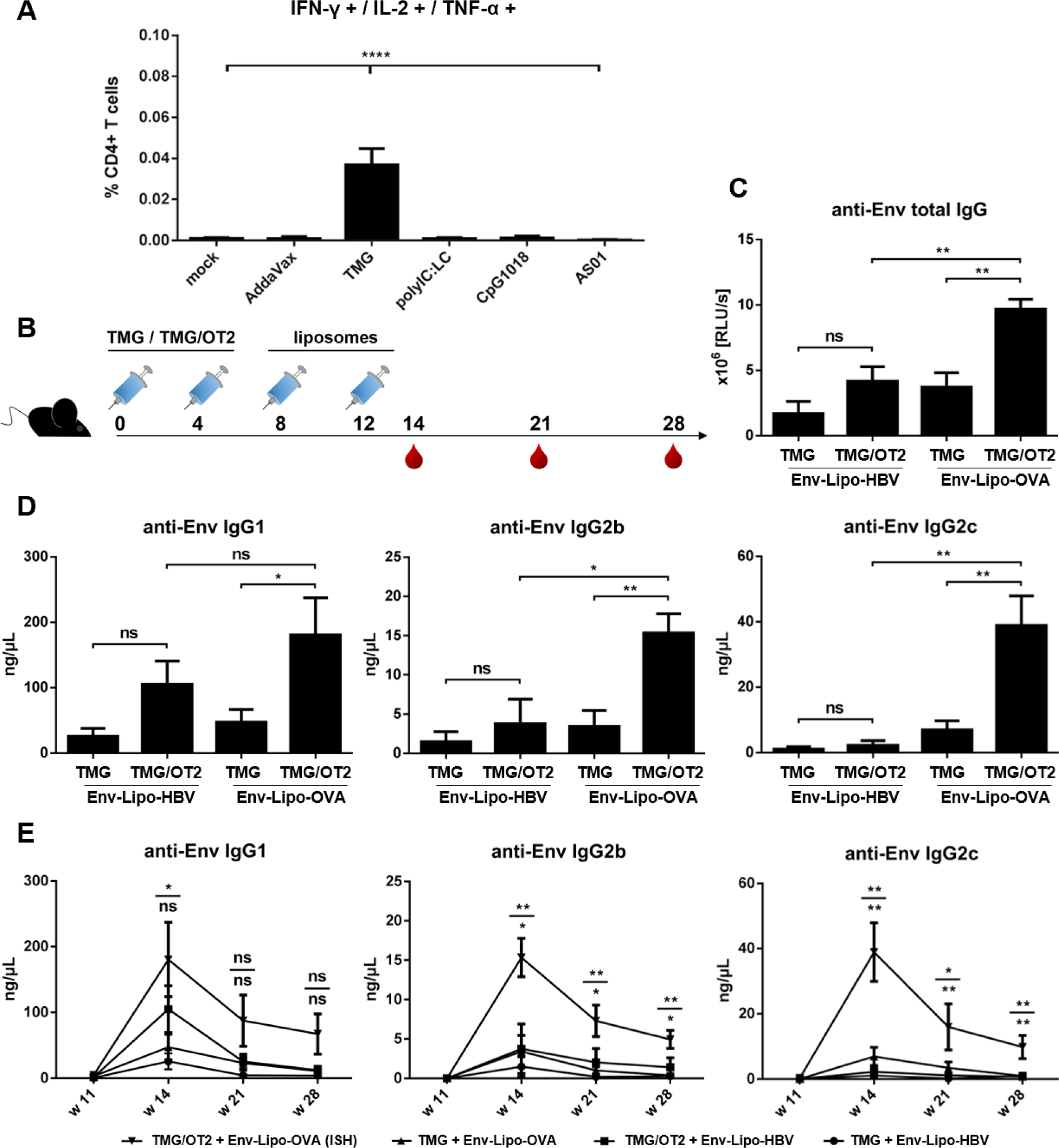
OVA-mediated ISH with cationic T helper liposomes. **(A**) Adjuvant screening for the elicitation of OT2-specific CD4+ T cells. BL6 mice were immunized twice (w0, w4) with 5 μg OT2 peptide mixed with various adjuvants in ratios according to the manufacturers’ recommendations. The splenocytes were isolated in week 6, restimulated with OT2 peptide and analyzed by flow cytometry. The columns ± SEM demonstrate the percentage of OT2-specific, polyfunctional (IFN-γ+, IL-2+, TNF-α+) CD4+ T helper cells per group. All groups were statistically compared via one-way ANOVA with Tukey’s post-hoc test. **** p < 0.0001. **(B)** BL6 mice (n = 6) were primed twice (w0, w4) with 5 μg OT2 peptide mixed 1:1 (w:v) with TMG or TMG alone and were boosted with either cationic Env-Lipo-HBV or Env-Lipo-OVA T helper liposomes in w8 and w12 (a total of 3 μg Env per animal and time point). Blood was taken in weeks 14, 21 and 28. **(C)** anti-Env total IgG endpoint levels (in RLU/s) in week 14. The columns ± SEM show the means of each indicated group. Significant differences between groups were evaluated by Mann Whitney non-parametric *t* test. ** *p <* 0.005. **(D)** anti-Env IgG subtype responses in week 14. The columns ± SEM represent the mean anti-Env IgG1 (left), IgG2b (center) and IgG2c (right) serum concentrations in ng/μL per group. Mann-Whitney tests were performed for statistical analyses. * *p <* 0.05; ** *p <* 0.005. **(E)** Long-term kinetics of elicited immune responses. The graphs demonstrate the progression of anti-Env IgG1, IgG2b and IgG2c levels (in ng/μL) in immunized mice up to week 28. Shown are the means ± SEM per group and timepoint. Statistics were performed by Mann-Whitney t tests. * *p <* 0.05; ** *p <* 0.005. Upper statistics compare priming regimens (TMG/OT2 + Env-Lipo-OVA vs. TMG + Env-Lipo-OVA). Lower statistics compare T cell help (TMG/OT2 + Env-Lipo-OVA vs. TMG/OT2 + Env-Lipo-HBV).

Our first aim was to elicit ISH with T helper liposomes, that encapsulated OT2 as a model peptide (Env-Lipo-OVA). We performed preliminary experiments with lentiviral VLPs incorporating a Gag-OT2 fusion protein (Env-VLP-OVA). In a first trial, we primed mice with OT2 peptide supplemented with alum as an adjuvant and boosted them with Env-VLP-OVA. Surprisingly, we did not see any ISH effects compared to the control animal group primed with alum alone (Fig. S5A). Therefore, we performed a screening to identify the most efficient adjuvant for the induction of OT2-specific CD4+ T cell responses. Mice were immunized twice in a 4-week interval with 5 μg OT2 peptide supplemented with various adjuvants (AddaVax^™^ [37], TiterMaxGold^®^ [28], polyIC:LC [38], CpG1018 [39] and AS01 [40]) as recommended by the manufacturers’ instructions and previous publications. We harvested the splenocytes in week 6 and performed an ICS after restimulating the cells *in vitro* with soluble OT2 peptide. We identified TiterMaxGold^®^ (TMG) to be the only adjuvant that mediated an induction of polyfunctional (IFN-γ+, IL-2+, TNF-α+) OT2-specific T helper cells (Fig. 5A). In a preliminary *proof-of-principle* trial, we primed BL6 mice twice with 5 μg OT2 peptide supplemented 1:1 (w:v) with TMG (TMG/OT2). As a prime control, another group of mice was primed with 5 μg HBsAg_17–40_ peptide mixed with TMG (TMG/pHBsAg). After two booster immunizations with Env-VLP-OVA, we did see significantly elevated anti-Env total IgG, IgG1 and IgG2c levels in the ISH group two weeks after the last nanoparticle immunization (Fig. S5A). Alternatively, we tested the induction of ISH by priming with ovalbumin-encoding DNA (OVA-DNA). This immunization regimen also resulted in a stronger induction of anti-Env IgG subtype responses compared to a control group primed with empty vector. However, in this setup the differences in the serum concentrations of the Env-specific IgG subtypes were not significantly elevated and were lower compared to the TMG-based regimen (51.0 ng/μL [TMG/OT2] vs. 8.7 ng/μL [OVA-DNA] for IgG1; 55 ng/μL [TMG/OT2] vs. 7.5 ng/μL [OVA-DNA] for IgG2c). We further evaluated the long-term kinetics (w14 – w21) of antibody responses in the TMG/OT2- and TMG/pHBsAg-primed mice (Fig. S5B). While the Env-specific IgG subtype concentrations in the non-ISH group had declined to background levels in week 21, those of the ISH group remained significantly increased (20.4 ng/μL IgG1, 27.2 ng/μL IgG2c). The data demonstrate that OT2-mediated ISH has effects on both Env-specific IgG1 and IgG2c subtype levels with beneficial long-term kinetics and that an TMG/OT2 peptide prime resulted in improved outcomes in comparison to an OVA-DNA prime.

We used these insights for the induction of ISH with cationic Env-Lipo-OVA. BL6 mice were primed twice with TMG/OT2 or TMG alone and boosted with Env-Lipo-OVA or Env-Lipo-HBV (Fig. 5B). As expected, the ISH group (TMG/OT2 + Env-Lipo-OVA) showed significantly increased anti-Env total IgG levels two weeks after the second liposomal boost (w14, Fig. 5C) compared to all other groups. Furthermore, the ISH group had significantly elevated anti-Env IgG1, IgG2b and IgG2c subtype responses compared to the prime control groups. Harnessing ISH, we induced mean endpoint concentrations of 180.7 ng/μL IgG1, 15.4 ng/μL IgG2b and 38.9 ng/μL IgG2c in week 14 (Fig. 5D). Notably, the Env-specific IgG1 serum concentrations in the control groups were rather strong, especially with TMG/OT2 + Env-Lipo-HBV (105.2 ng/μL IgG1), probably due to depot effects caused by TMG. The effects of ISH resulted in more favorable long-term kinetics of Env-specific antibody responses (Fig. 5E). In particular, the Th1 responses (IgG2b and IgG2c) were significantly elevated up to week 28 in the ISH groups compared to the control groups, which have dropped to background levels at this time point.

Thus, we showed that cationic T helper liposomes with a model peptide (OT2) could be utilized to induce ISH effects, that promoted both Env-specific Th1 and Th2 responses in mice. Nevertheless, the phenotypic outcomes in terms of endpoint IgG subtype concentrations were more pronounced for Th2 responses and less pronounced for Th1 responses compared to immunizations with Env-VLP-OVA (Fig. S5).

**Fig. 6.**
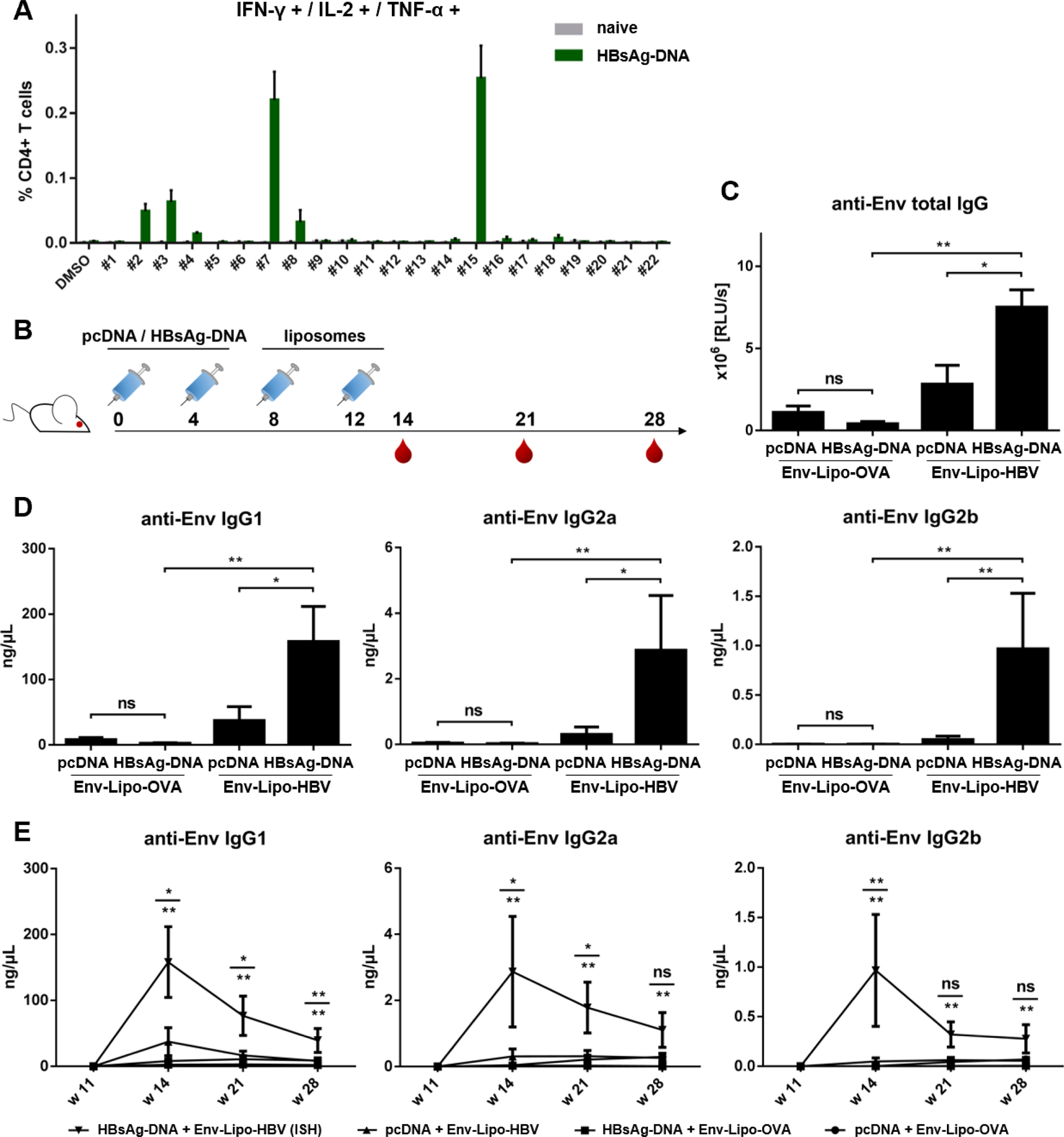
HBV-mediated ISH with cationic T helper liposomes. **(A**) HBsAg peptide screening. BALB/c mice were immunized twice (w0 and w4) with 30 μg DNA coding for the HBV surface antigen (HBsAg). Splenocytes from immunized and naïve mice (n = 6) were isolated in week 6 and restimulated with single epitopes from a peptide library spanning the whole HBsAg aa sequence (peptide #1 - #22) or with DMSO as a mock control and analyzed by flow cytometry. Shown are the mean percentages ± SEM of polyfunctional (IFN-γ+, IL-2+, TNF-α+), peptide-specific CD4+ T helper cells per group. **(B)** BALB/c mice (n = 6) were primed twice (w0, w4) with 30 μg HBsAg-DNA or empty vector (pcDNA) by electroporation and were boosted i.m. either with cationic Env-Lipo-OVA or Env-Lipo-HBV T helper liposomes in w8 and w12 (3 μg Env per animal and time point). Blood was taken in weeks 14, 21 and 28. **(C)** anti-Env total IgG endpoint levels (in RLU/s) at week 14. The columns ± SEM demonstrate the means of each indicated group. Significant differences between groups were evaluated by Mann Whitney non-parametric *t* test. * *p <* 0.05, ** *p <* 0.005. **(D)** anti-Env IgG subtype responses in week 14. The columns ± SEM represent the mean anti-Env IgG1 (left), IgG2a (center) and IgG2b (right) serum concentrations in ng/μL per group. Mann-Whitney tests were performed for statistical analyses. * *p <* 0.05, ** *p <* 0.005. **(E)** Long-term kinetics of elicited immune responses. The graphs demonstrate the progression of anti-Env IgG1, IgG2a and IgG2b levels (in ng/μL) in immunized mice up to week 28. Shown are the means ± SEM per group and timepoint. Statistics were performed by Mann-Whitney t tests. * *p <* 0.05; ** *p <* 0.005. Upper statistics compare priming regimens (HBsAg-DNA + Env-Lipo-HBV vs. pcDNA + Env-Lipo-HBV). Lower statistics compare T cell help (HBsAg-DNA + Env-Lipo-HBV vs. HBsAg-DNA + Env-Lipo-OVA).

We further intended to induce ISH effects with T helper liposomes, that encapsulated an epitope derived from a heterologous pathogen. Since the global coverage of HBV vaccination largely improved during the last decade, pre-existing HBV-specific T helper cells have become of interest for a potential clinical ISH trial. To provide a *proof-of-principle* experiment in mice, we screened a peptide library (n = 22) covering the entire HBsAg aa sequence to identify epitopes that efficiently restimulate T cell responses from immunized animals. To this end, we immunized BALB/c mice twice (w0 and w4) with 30 μg HBsAg-encoding DNA. The splenocytes of these animals and of a naïve control group were isolated in w6 and restimulated with each of the 22 peptides from the library (Fig. 6A). Unstimulated controls incubated with DMSO only as well as the splenocytes from naïve mice were used as negative controls. The highest percentage of restimulated, polyfunctional CD4+ T cells was seen in samples incubated with peptide #7 and #15. A smaller degree of restimulation was mediated by peptides #2, #3 and #8. All other tested peptides induced either insufficient or no restimulation of HBsAg-specific CD4+ T cells. Based on these findings, we performed a preliminary trial with lentiviral T helper VLPs incorporating a fusion construct of Gag and peptide #15 (Env-VLP-HBV). BALB/c mice were primed twice (w0 and w4) with either HBsAg-encoding DNA or empty vector (pcDNA) by electroporation and were boosted i.m. in week 8 and week 12 with Env-VLP-HBV (Fig. S6). Despite elevated anti-Env IgG responses in the ISH group (HBsAg-DNA + Env-VLP-HBV), the differences compared to the mock prime group were only significant for IgG2a. The mean serum concentrations were 26.3 ng/μL for IgG1 and 37.5 ng/μL for IgG2a. In favor of an optimal electrostatic encapsulation efficiency into cationic liposomes, we designed a peptide that covers parts of the aa sequence of the reactive cluster peptides #2 and #3 (HBsAg_17–40_) and generated Env-Lipo-HBV (Fig. 1). BALB/c mice were immunized twice with pcDNA or HBsAg-DNA and boosted in week 8 and week 12 with Env-Lipo-OVA or Env-Lipo-HBV (Fig. 6B). The analysis of Env-specific total IgG endpoint levels in week 14 showed a significant increase in the ISH group (HBsAg-DNA + Env-Lipo-HBV) compared to the control groups (Fig. 6C).

Via ISH the mean serum concentration of anti-Env IgG1 was 158.2 ng/μL in week 14. Even though Th1 responses were significantly elevated in the ISH group, the mean serum concentrations of both Env-specific IgG2a and IgG2b were below 10 ng/μL (Fig. 6D). Nevertheless, the long-term kinetics of Env-specific subtype antibodies improved by ISH were comparable to those of the OVA-mediated trial. In mice that received ISH modulation the mean serum concentrations of Env-specific IgG1 were 76.7 ng/μL in week 21 and 39.3 ng/μL in week 28, which was a strongly significant difference compared to all control groups that have dropped to background levels at these time points (Fig. 6E).

In summary, the antibody responses induced by cationic T helper liposomes encapsulating a CD4+ T cell epitope derived from a widely used human vaccine can be modulated by ISH. Thus, cationic T helper liposomes are a promising tool for the modulation of humoral immune responses in the context of various pathogens and clinical setups.

## 4. Discussion

The advantages of nanoparticles over soluble proteins for vaccine formulas have become a steadily increasing focus of HIV vaccine research during the last two decades [14]. The repetitive display of Env antigens on the nanoparticle surface leads to an improved BCR crosslinking resulting in a stronger B cell activation [20–22,41,42]. Moreover, in preclinical studies the immunization of animals with nanoparticles correlated with stronger elicitation of cross-neutralizing immune responses against HIV, HCV and LASV [19,43–46], even though one study did not observe an improvement of HIV-1 neutralization in rhesus macaques immunized with liposomes compared to soluble Env trimers [12]. How these different immunological outcomes were affected by i) the type of nanoparticle, ii) the choice of antigen and iii) the coupling mechanism is not yet fully understood [47].

The cationic liposomes presented in this study were conjugated with a tag-free mechanism harnessing the negative zetapotential of Env trimers for electrostatic interactions with the liposomal surface followed by EDC/Sulfo-NHS chemistry [33]. Tag-free functionalization with surface antigens is common for self-assembling nanoparticle systems, where Env is genetically fused to a structural component [48–50]. However, reports about tag-free conjugation of Env trimers in an orthogonal manner to preformed nanoparticles are rare. Ringe *et al*. harnessed EDC/Sulfo-NHS chemistry to conjugate iron oxide nanoparticles with Env trimers, but utilized a C-terminal KG4-tag that is targeted by the NHS ester [51]. Streif *et al.* recently used the same coupling strategy as in the current study to immobilize tag-free SARS-CoV-2 receptor binding domain (RBD) on the surface of liposomes [52]. We performed a tag-free coupling mechanism in order to support GMP-grade clinical applications with our T helper liposomes in the future. Even though the coupling mechanism is not site-specific, we observed liposomes with multiple propeller-like Env trimer structures on the surface [53]. Furthermore, immunogold staining and flow cytometry with liposomes revealed binding of trimer-specific antibodies to the liposomes (Fig. S1). These data are consistent with an ELISA assay published elsewhere that showed minor loss of conformation of the SUFO.664 trimers on the surface of the cationic liposomes [33]. Interestingly, in this previous study a mean of 4–5 coupled Env trimers per liposome was calculated by biochemical methods. In contrast to that, the EM images of the current study show a range of 20–100 Env trimers per cationic liposome. Generally, the tag-free coupling mechanism was less efficient than tag-based approaches, however, a high valency of displayed antigens bears the risk of triggering an extrafollicular B cell response, so that the required germinal center reaction might be dampened [54]. Tokatlian *et al.* (2018) tested liposomes conjugated with more than 300, 150 or 50 Env trimers for their ability to activate VRC01 B cells *in vitro* and to trigger a germinal center reaction *in vivo.* The authors observed the strongest B cell activation with the highest valency of Env and immunizations with liposomes generally promoted the formation of antigen-specific germinal center B cells compared to vaccinations with soluble trimers [55]. On the contrary, Veneziano *et al.* (2020) reported that a display of only 5 antigens on DNA origami nanoparticles was sufficient to stimulate B cell responses [56].

In the course of the last decade, we utilized the unique features of nanoparticles to encapsulate heterologous T helper epitopes. After surface functionalization of these T helper nanoparticles with the antigen of interest, we harnessed a loophole in the immune system to recruit pre-existing, heterologous T cell responses, that could provide ISH for antigen-specific B cells upon immunization [23,24,27]. Besides a more rapid and increased humoral immune response, the effects of ISH may alter the IgG subtype ratio, which could have beneficial effects for Fc effector functions [25,26]. T helper nanoparticles require the quantitative encapsulation of heterologous epitopes and the display of the antigen of interest on the surface. We carefully improved and evaluated our inorganic and organic T helper nanoparticle designs over the years [21,29].

We have switched from an anionic liposome system to a cationic one not only due to the tag-free electrostatic interactions with negatively charged Env trimers, but also because many studies describe a preferential uptake of cationic nanoparticles by APCs [57–60]. In fact, in this study cationic T helper liposomes mediated stronger activation and proliferation of OT2-specific CD4+ T cells in an *in vitro* ISH setup compared to anionic liposomes (Fig. 2). The size and PDI of our cationic T helper liposomes allows them to enter and pass lymphatic vessels [17,42]. The *in vivo* stability of T helper nanoparticles is crucial for a successful induction of ISH. In a previous study, we have demonstrated that OT2 peptides encapsulated into liposomes were efficiently presented by DCs in the spleen and inguinal lymph nodes of mice after intramuscular immunization [29]. The current study provides evidence via an adoptive transfer trial that T helper liposomes reach secondary lymphoid organs and lead to proliferation of Env-specific B cells *in vivo* after intramuscular injection (Fig. 4). Since soluble Env trimers are not able to effectively activate cognate B cells, our results suggest that the tag-free Env conjugation to the liposomal surface is stable *in vivo* and that the liposomes maintain their structural features for a repetitive display of the surface antigen. While the liposomes reach the inguinal lymph nodes probably through lymphatic vessels, this route cannot be considered for the spleen, since this organ is not directly connected to the lymphatic system [61]. Thus, the T helper liposomes must have reached the spleen via the blood stream. One possible explanation is that small blood vessels are penetrated during intramuscular immunization and some of the injected liposomes enter the bloodstream. A similar observation was made during the evaluation of the licensed COVID-19 vaccines based on lipid nanoparticles encapsulating mRNA. Here, expression of the SARS-CoV-2 S antigen was not just detected in the muscle tissue at the injection site, but in multiple organs throughout the vaccinees’ bodies [62]. Low-affinity IgM in the bloodstream may bind to nanoparticles with repetitive surface antigens and form immune complexes, which are transferred to FDCs at the site of germinal centers [42]. Trafficking of nanoparticles conjugated with highly-glycosylated Env trimers to FDCs can further be mediated by mannose-binding lectins (MBLs) [63]. In summary, the size and injection route of nanoparticles are decisive parameters for the interaction with the immune system. To elicit ISH effects the T helper nanoparticles must be drained to secondary lymphoid organs and taken up by cognate B cells that subsequently recruit heterologous T helper cells.

We carefully conducted preliminary ISH trials with T helper VLPs as a reliable reference for the recruitment of heterologous T cell responses, before we performed analogous experiments with cationic T helper liposomes (Fig. 5 and S5). OT2 was utilized as a model T helper epitope. Unexpectedly, a major hurdle was to induce OT2-specific T helper cell responses. First, we used OT2 peptide supplemented with alum, since we had good experience in the induction of Tetanus-specific T cell responses with this adjuvant, but did not detect ISH effects. Nevertheless, Hills *et al.* (2017) reported the modulation of the humoral immune responses against the *Plasmodium falciparum* CSP antigen utilizing T helper liposomes encapsulating OT2 [28]. Here, OT2 T cell responses were induced by the adjuvant TiterMaxGold^®^ (TMG). In the present study, we performed a screening of multiple adjuvants in combination with OT2. In fact, we found that TMG is the sole adjuvant that mediated the induction of OT2-specific T helper cell responses (Fig. 5A). The phenotype of the OT2-mediated ISH with both T helper liposomes and VLPs was consistent with the IgG subtype modulations observed by Hills *et. al.* [28]. Both Env-specific Th2 (IgG1) and Th1 (IgG2b, IgG2c) responses were significantly elevated in the ISH group (TMG/OT2 + Env-Lipo-OVA; Fig. 5). Beside ISH, we also observed pronounced anti-Env IgG1 responses in the control groups, which might be attributed to unique features of TMG, because the water-in-oil adjuvant promotes Th2 immune responses [64]. Furthermore, TMG builds depots at the injection sites, which last up to 28 days and might have directly acted on Env after the first liposomal boost (w8) [65]. We tried to circumvent this issue by performing TMG/OT2 prime immunizations in the left hind leg of mice and the liposomal boosts in the right hind leg to avoid possible interactions between liposomes and TMG/OT2 depots. However, we did not see any induction of ISH with this regimen (data not shown). Nevertheless, this is an indirect indication that the essential T cell / B cell interactions leading to ISH may majorly take place in the germinal centers of draining lymph nodes at the injection sites and not in all secondary lymphoid organs across the body. Notably, we performed the OT2-mediated ISH trials in BL6 mice, since OT2 is known as an immunodominant peptide for this strain [66]. However, priming mice with OVA-encoding DNA resulted in less pronounced ISH effects (Fig. S5). In fact, it is described elsewhere that proteasomal degradation of unmodified OVA in BL6 mice does not result in the presentation of OT2 peptides on MHC-II molecules. Probably, overlapping peptides with less affinity to the OT2-specific T cell receptors (TCRs) are produced *in vivo* [67]. These data might explain the weak ISH phenotypes observed with OVA-DNA-primed mice.

On the contrary, HBV-associated liposomal ISH trials were done in BALB/c, because the screening of the HBsAg peptide library was more pronounced in this mouse strain (Fig. 6A). We observed the strongest reactivity of HBsAg-specific T cells after restimulation with HBsAg peptide #15. However, a preliminary ISH trial with lentiviral VLPs incorporating peptide #15 resulted in insufficient ISH effects (Fig. S6). Improper peptide processing from the Gag/#15 fusion protein might be an explanation for this. Moreover, the ICS assay (Fig. 6A) predominantly indicated the amount of reactivated cells, but not the affinity of TCRs. TCR affinity is crucial for the generation of effective immune responses, particularly in the context of germinal center reactions. The strength of the TCR/peptide-MHC interaction determines the level of T cell help provided for the B cells. T follicular helper (Tfh) cells with high-affinity TCRs have been shown to provide effective B cell help, which promoted germinal center reactions and increased somatic hypermutation [54,68]. Thus, peptide/TCR affinity probably plays a more important role for ISH than overall peptide-specific CD4+ T cell frequency. For the liposomal ISH approach, we chose an overlapping peptide from a reactive cluster (HBsAg library peptide #2 and #3) for encapsulation into our cationic T helper liposomes (HBsAg_17–40_). Consistent with the preliminary peptide screening, we conducted this immunization trial in BALB/c mice and, therefore, analyzed anti-Env IgG2a responses as the main Th1-like IgG subtype [69]. Mice were primed with HBsAg-encoding DNA or empty vector and boosted with Env-Lipo-OVA or Env-Lipo-HBV. The ISH group (HBsAg-DNA + Env-Lipo-HBV) demonstrated a strong and significant upregulation of anti-Env IgG1. Th1 responses were also significantly increased in the ISH group, but the total serum concentrations of Env-specific IgG2a and IgG2b antibodies were lower compared to IgG1. Nevertheless, these results pave the way for further trials using licensed HBV vaccines for the prime immunization. Notably, ISH also significantly improved the long-term Env-antibody kinetics in both liposomal trials (Fig. 5E, Fig. 6E) as well as in the VLP trial (Fig. S5B) compared to all control groups.

We could not elicit *in vivo* ISH effects with an early generation of our T helper liposomes (Fig. S4), that were functionalized with a dense array of Env spikes, but encapsulated less than ten T helper epitopes per liposome, even though these liposomes were able to induce *in vitro* ISH effects [29]. This observation raises the question, whether the crucial parameter for *in vivo* ISH induction is the amount of encapsulated T helper epitopes alone and whether the number of displayed antigens of interest plays a subordinate role. Further experiments with a titration of peptides and Env on T helper nanoparticles need to be performed to address this issue in the future.

Beside the liposomal ISH described by us and Hills *et al.* [28], others elicited ISH effects utilizing MPER-functionalized liposomes that encapsulated LACK1 or HIV30 T helper peptides. Even though the authors did not investigate anti-MPER IgG subtype responses, the induction of ISH resulted in a significantly increased MPER-specific total IgG response and effective germinal center formation [70].

Notably, we did not perform neutralization assays with the murine sera, since wildtype mice cannot effectively neutralize tier-2 strains of HIV-1 [71,72]. Nevertheless, the immunization regimen may be applied to transgenic mice with humanized immunoglobulins in the future [73,74]. The translation of ISH trials into higher animals or clinical applications comes with hurdles. The suitability of the nanoparticles for GMP production was overcome with the establishment of a tag-free Env conjugation mechanism [33]. Another challenge, the breadth of the coverage of T cell restimulation among vaccinees in non-inbred animal systems or humans, will need to be addressed by further screenings of T helper peptide libraries or encapsulation of unprocessed proteins.

## 5. Conclusions

Taken together, we present a novel, tag-free nanoparticle system that can be utilized for the quantitative and qualitative modulation of HIV-specific humoral immune responses by harnessing pre-existing T cell responses via ISH. These features render T helper liposomes promising vaccine candidates for future clinical vaccine trials in the context of HIV and other pathogens.

## Supplementary Material

Supplement

## Data Availability

Data will be made available on request.
