# Supplementary material for: Modulation of immune responses to liposomal vaccines by intrastructural help
Source: Eur J Pharm Biopharm. Author manuscript; Available in PMC 2024 Feb 16. (PMC10872448; doi:10.1016/j.ejpb.2023.10.003)
Supplement: Supplement [file NIHMS1942595-supplement-Supplement.docx]

**Supplementary Material**

**Figure S1**


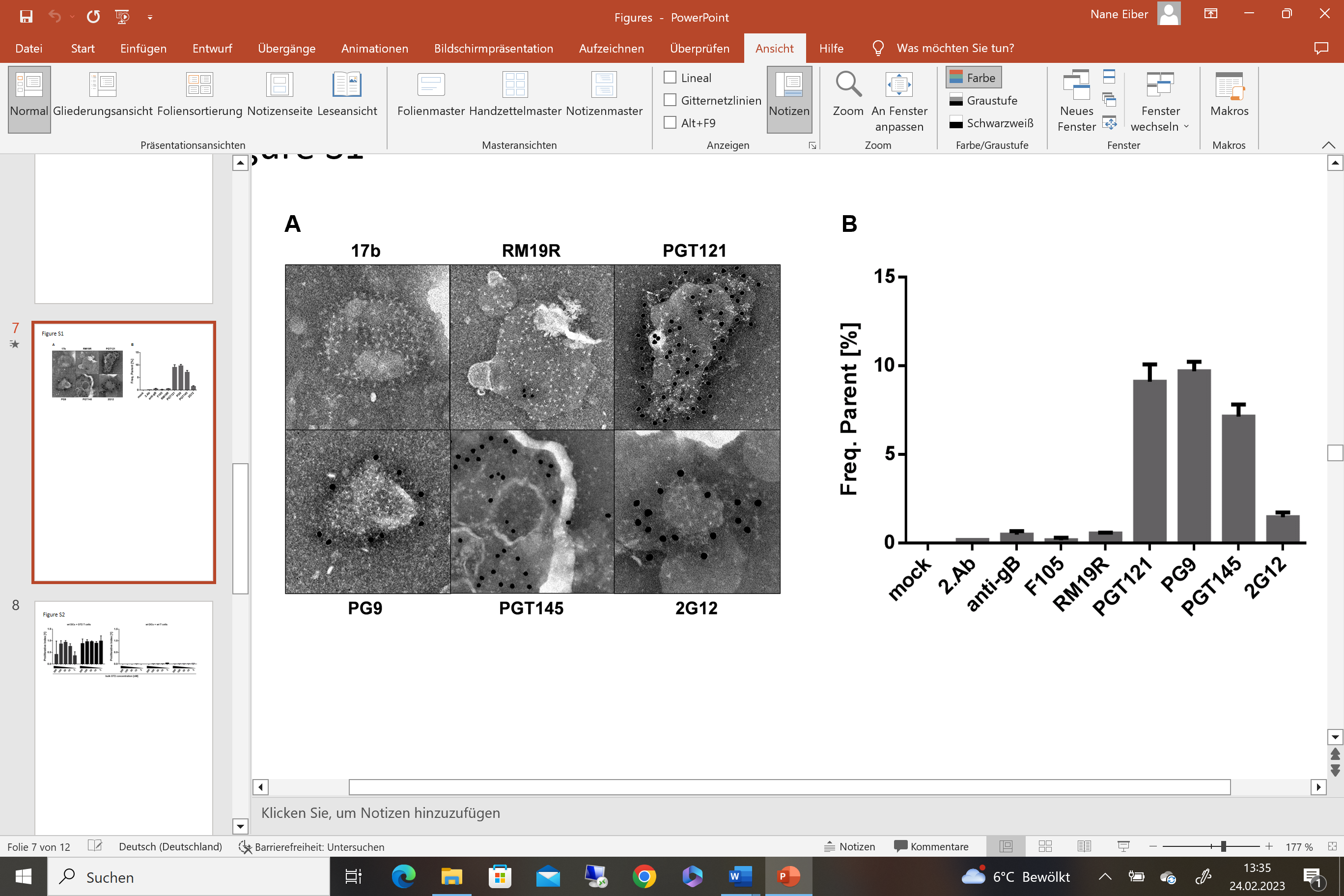


**Figure S1: Biophysical characterization of cationic T helper liposomes. (A)** Immunoelectron microscopy. Cationic T helper liposomes were immobilized on a TEM grid and labelled with conformation-specific monoclonal antibodies (3 µg/mL): 17b (CD4i), RM19R (trimer base), PGT121 (gp120 interface), 2G12 (glycan patch), PG9 and PGT145 (both trimer apex) all directly conjugated to gold beads with a diameter of 10 nm. After immunolabeling, the samples were washed to remove free antibodies and subsequently analyzed by TEM. Black dots represent the gold bead-coupled antibodies bound to Env on the surface of the liposomes. **(B)** Liposomal surface stain. Cationic T helper liposomes were stained with a panel of monoclonal antibodies (1 µg/mL). F105 specific for the open conformation of Env and the hCMV-specific 27-287 anti-gB antibody were used as negative and isotype control, respectively. The samples were further stained with fluorescent anti-human IgG secondary antibody (1 µg/mL) and analyzed by flow cytometry.

**Figure S2**


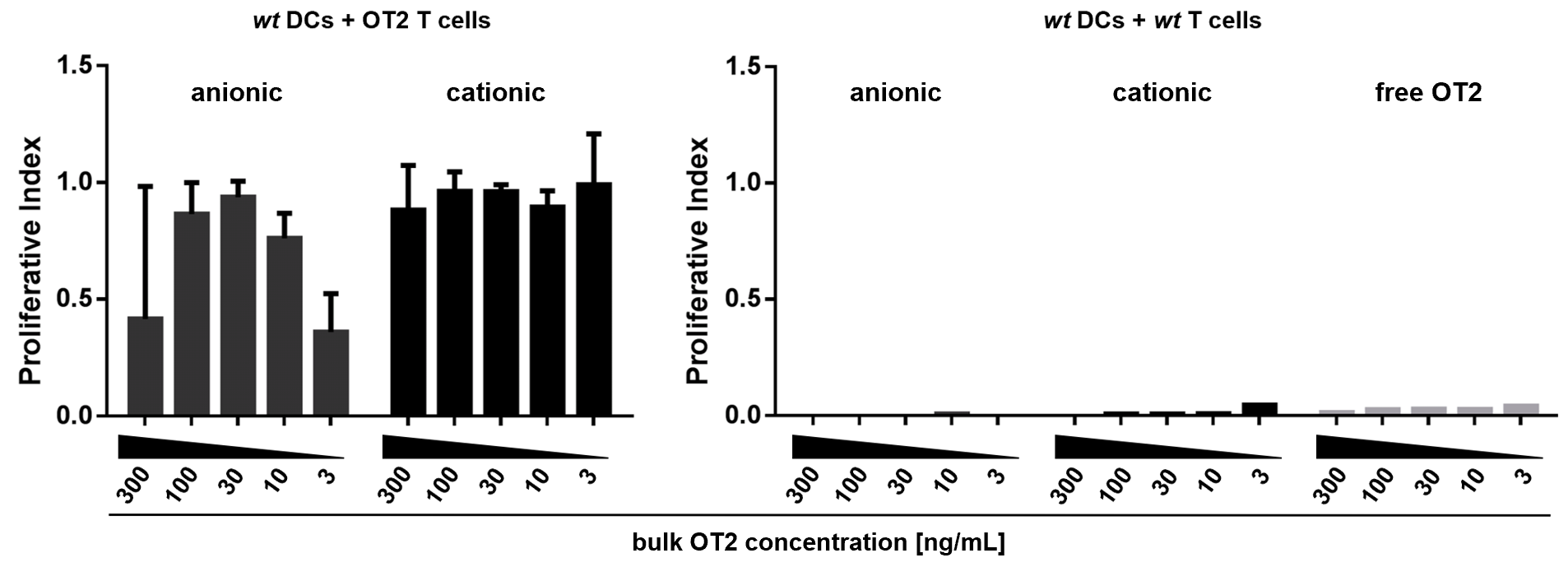


**Figure S2: MHC-II-mediated presentation of liposomal peptides.** OT2-specific or *wt* T helper cells and *wt* DCs were isolated and purified by MACS. The T cells were stained with 7.5 µM CFSE. Subsequently, co-cultures of 1 x 10^5^ T cells and 1 x 10^5^ DCs, respectively, were seeded in U-bottom 96-well plates. The co-cultures were incubated for 3 days in the presence of anionic or cationic OT2-encapsulating liposomes or free OT2 peptide in a dilution series normalized to the bulk concentration of OT2 (300 – 3 ng/mL). Proliferation was measured based on the CFSE distribution pattern. The percentage of proliferation of CD4+ T cells in each sample was divided by the percentage of OT2 T cell proliferation in the presence of corresponding concentrations of free OT2 peptide (proliferative index). Shown are the bars ± SEM of two independent experiments (*wt* DCs + OT2 T cells) and the bars of one control experiment (*wt* DCs + *wt* T cells).

**Figure S3**


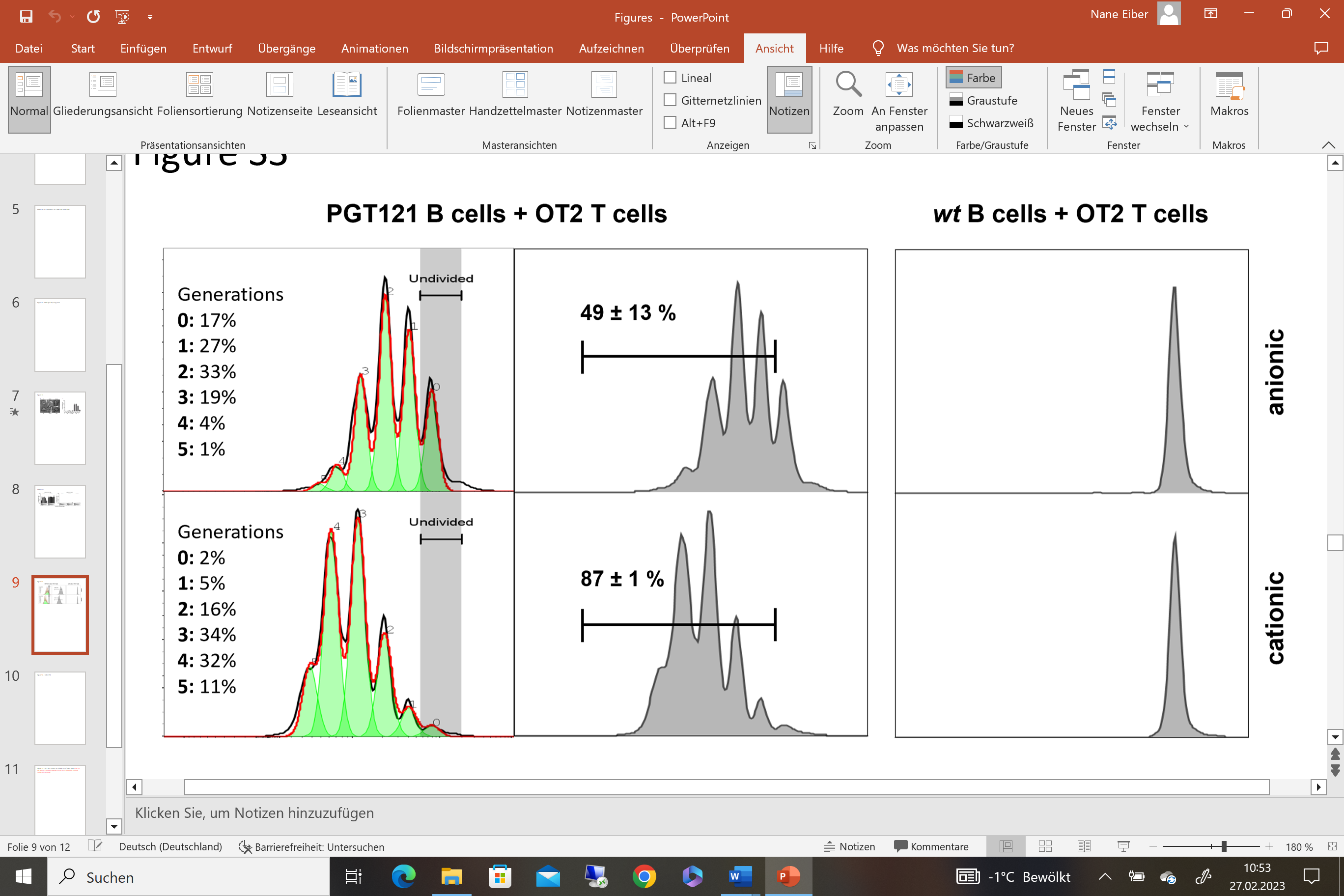


**Figure S3: Analysis of T cell proliferation patterns.** 1 x 10^5^ CFSE-labeled CD4+ T cells (OT2-specific) were co-cultures with 1 x 10^5^ B cells (*wt* or Env-specific) for 3 days in the presence of 8 ng/mL bulk Env on anionic or cationic T helper liposomes. Shown are the control culture with *wt* B cells (right panel), the proliferation curves with the indicated gates for proliferating CD4+ cells (center panel) and a calculation of the percentages of undivided (Generation 0) and proliferated cells per generation using the FlowJo Proliferation Platform™ (left panel).

**Figure S4**


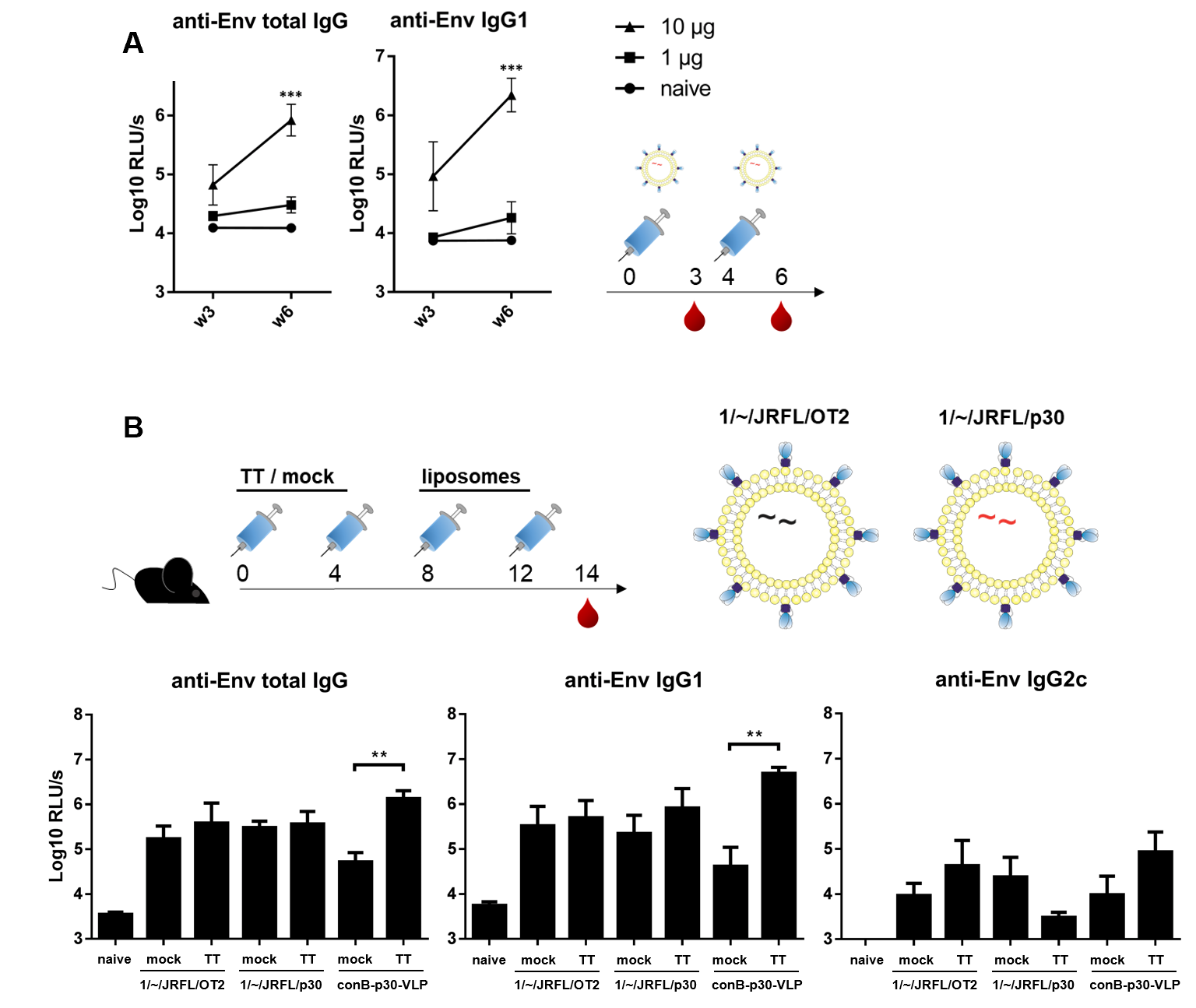


**Figure S4: Intrastructural help trials with first-generation T helper liposomes. (A)** Liposomal dose finding. BALB/c mice were immunized twice (w0, w4) i.m. with first-generation T helper liposomes. The immunization doses per group (n = 4) were adjusted to a total amount of 1 µg or 10 µg Env (JRFL) on liposomes per mouse. A third group (n = 2) remained naive. Blood was taken in week 3 and week 6. The Env-specific humoral immune responses were analyzed by serum ELISA. Shown are the mean relative light units per second (Log10 RLU/s) for each experimental group and bleeding time point ± SEM. Significant differences in the immune response per timepoint were analyzed by one-way ANOVA comparison of all mouse groups with Tukey’s multiple comparison post-hoc test. *** *p* < 0.001. **(B)** ISH immunizations. BL6 mice (n = 6) were primed twice (w0, w4) with a licensed Tetanus vaccine (Tetanol®pur, TT) diluted 1:100 in PBS or PBS only (mock) and were boosted (w8, w12) with first-generation T helper liposomes (3 µg Env per mouse) encapsulating either OT2 as a control or the Tetanus Toxoid-derived p30 peptide (1/~/JRFL/OT2 and 1/~/JRFL/p30; nomenclature adapted from Damm *et al.,* 2022 ^32^). Two additional groups of BL6 mice were boosted with T helper VLPs (conB-p30-VLP) as a positive control. Shown are the Env-specific humoral immune responses in week 14 analyzed by serum ELISA with 1:100 diluted immune sera in Log10 RLU/s. The columns represent the mean ± SEM per group. For statistical analyses, groups that received a licensed vaccine prime were compared to the mock prime groups with identical nanoparticle boost by Mann-Whitney t-test. ** *p* < 0.005.

**Figure S5**


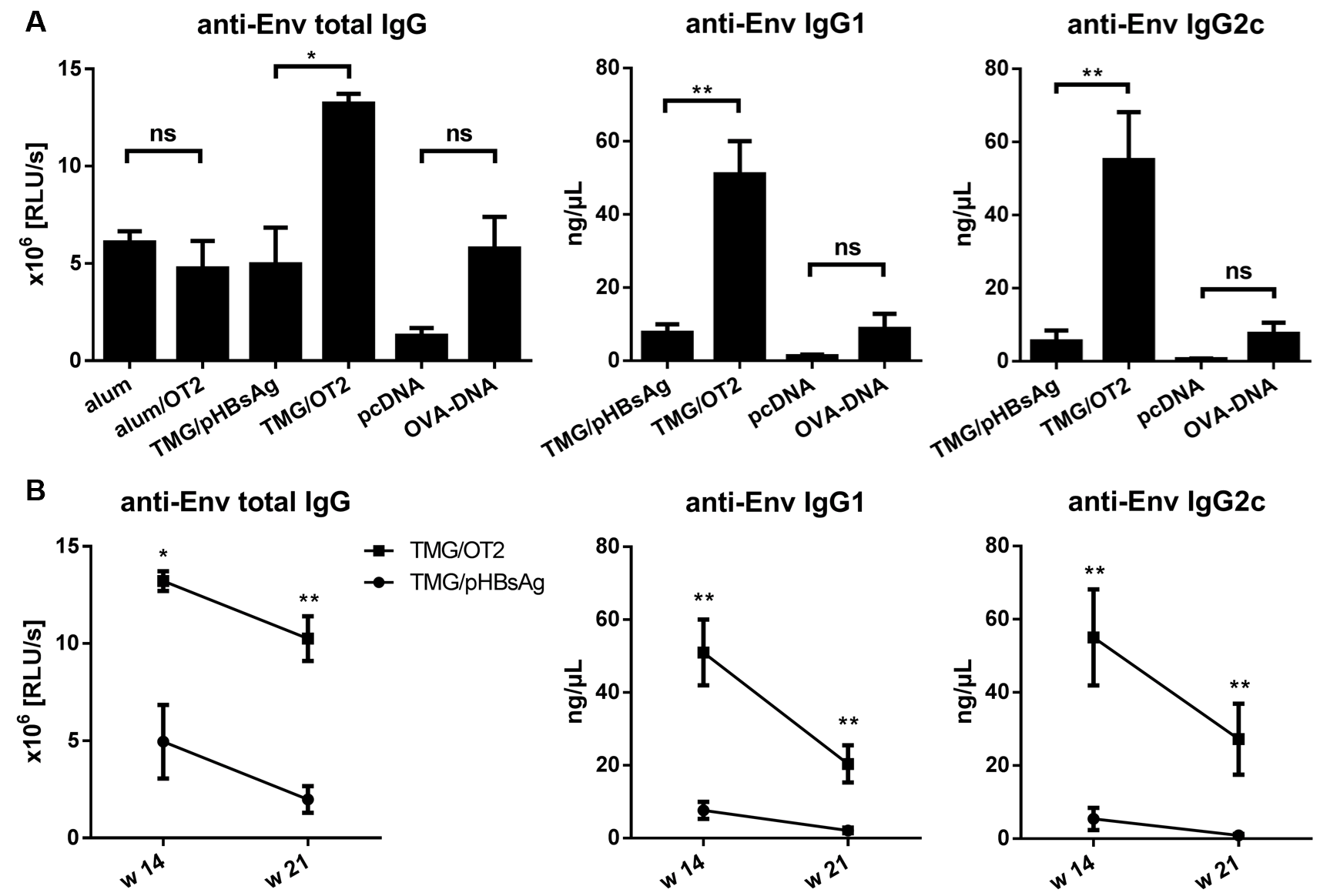


**Figure S5: OVA-mediated ISH with T helper VLPs. (A)** BL6 mice (n = 6) were primed twice with i) 5 µg OT2 peptide mixed 1:1 (w:v) with alum or alum alone, ii) 5 µg OT2 or an HBsAg peptide mixed with TiterMaxGold® (TMG/OT2, TMG/pHBsAg), iii) 30 µg empty vector (pcDNA) or OVA-encoding DNA (OVA-DNA). All mice were boosted twice i.m. with SUFO.750-OT2 T helper VLPs (Env-VLP-OVA). The columns ± SEM represent endpoint anti-Env total IgG (in RLU/s, left panel), IgG1 and IgG2c (in ng/µL serum, center and right panel) levels in week 14, two weeks after the last VLP immunization. Significant differences between ISH and respective mock prime groups were evaluated via Mann-Whitney non-parametric t test. * *p* < 0.05; ** *p* < 0.005. **(B)** Long-term anti-Env IgG kinetics in mice primed with TMG/OT2 or TMG/pHBsAg. The graphs ± SEM show the progression of endpoint total IgG, IgG1 and IgG2c between week 14 and week 21. Mann-Whitney tests were performed to compare ISH with non-ISH groups per time point. * *p* < 0.05; ** *p* < 0.005.

**Figure S6**


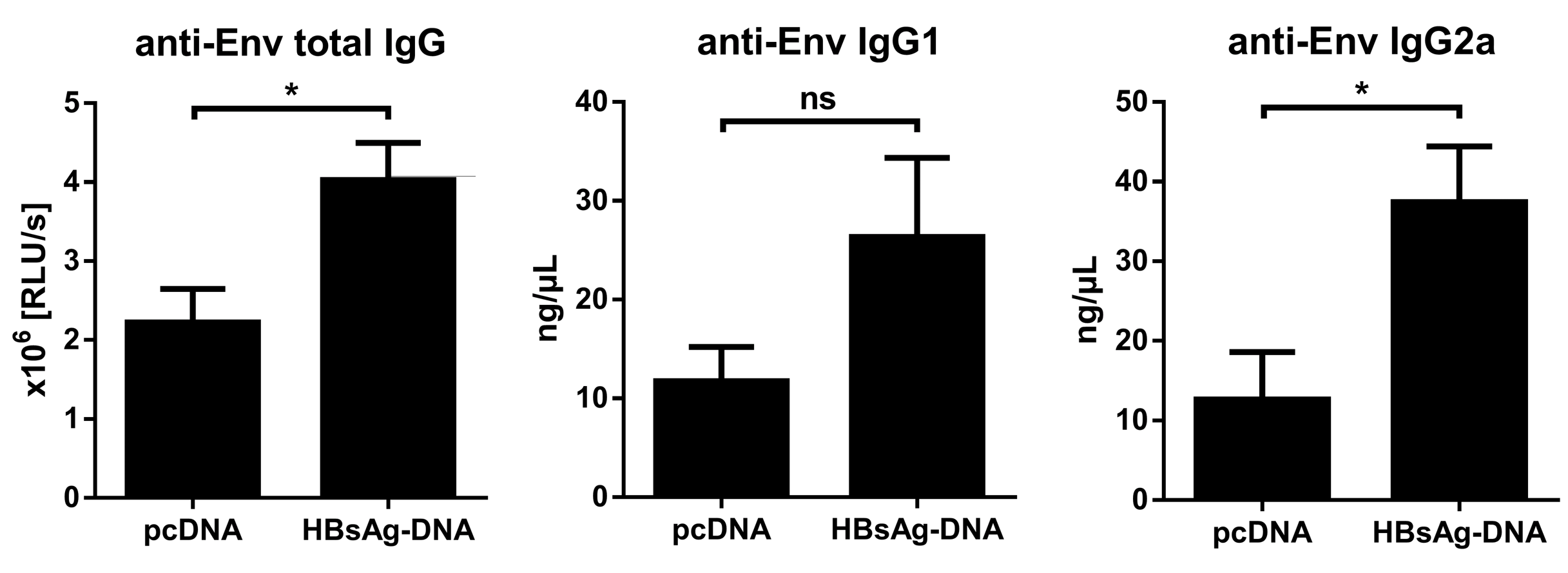


**Figure S6: HBV-mediated ISH with T helper VLPs.** BALB/c mice (n = 6) were primed twice with 30 µg empty vector (pcDNA) or HBsAg-encoding DNA (HBsAg-DNA) by electroporation. All mice were boosted twice i.m. with lentiviral T helper VLPs encapsulating HBsAg peptide #15 (Env-VLP-HBV). The columns ± SEM represent endpoint anti-Env total IgG levels (in RLU/s, left panel), IgG1 and IgG2a (in ng/µL serum, center and right panel) in week 14, two weeks after the last VLP immunization. Significant differences between ISH and respective mock prime groups were evaluated via Mann-Whitney non-parametric t test. * *p* < 0.05.
